# Spatial Comparisons of Mechanosensory Information Govern the Grooming Sequence in *Drosophila*

**DOI:** 10.1016/j.cub.2020.01.045

**Published:** 2020-03-05

**Authors:** Neil Zhang, Li Guo, Julie H. Simpson

**Affiliations:** 1 Department of Molecular, Cellular, and Developmental Biology and Neuroscience Research Institute, University of California, Santa Barbara, Santa Barbara, CA 93106, USA; 2 Lead Contact

## Abstract

Animals integrate information from different sensory modalities, body parts, and time points to inform behavioral choice, but the relevant sensory comparisons and the underlying neural circuits are still largely unknown. We use the grooming behavior of *Drosophila* melanogaster as a model to investigate the sensory comparisons that govern a motor sequence. Flies perform grooming movements spontaneously, but when covered with dust, they clean their bodies following an anterior-to-posterior sequence. After investigating different sensory modalities that could detect dust, we focus on mechanosensory bristle neurons, whose optogenetic activation induces a similar sequence. Computational modeling predicts that higher sensory input strength to the head will cause anterior grooming to occur first. We test this prediction using an optogenetic competition assay whereby two targeted light beams independently activate mechanosensory bristle neurons on different body parts. We find that the initial choice of grooming movement is determined by the ratio of sensory inputs to different body parts. In dust-covered flies, sensory inputs change as a result of successful cleaning movements. Simulations from our model suggest that this change results in sequence progression. One possibility is that flies perform frequent comparisons between anterior and posterior sensory inputs, and the changing ratios drive different behavior choices. Alternatively, flies may track the temporal change in sensory input to a given body part to measure cleaning effectiveness. The first hypothesis is supported by our optogenetic competition experiments: iterative spatial comparisons of sensory inputs between body parts is essential for organizing grooming movements in sequence.

## In Brief

Zhang et al. find that *Drosophila* covered with dust compare sensory inputs from mechanosensory bristles on different body parts during grooming. The ratio of anterior:posterior sensory input and its dynamics, rather than the rate of dust removal from the anterior, drives the anterior-to-posterior grooming sequence.

## INTRODUCTION

To organize a complex behavior, the nervous system needs to integrate sensory information from different types of sensory organs [1, 2] on different body parts [3, 4]. The absolute and relative sensory inputs to these body parts change over time [5, 6]. For example, dust can induce grooming behavior in *Drosophila melanogaster* [7]. When the fly is covered with dust, sensory organs all over the body are activated, but only one part is groomed at a time. The distribution of dust across the body changes as a result of grooming movements, so flies may be constantly re-assessing the relative amounts of dust. Therefore, *Drosophila* grooming provides a good platform to study the rules for integrating diverse time-varying sensory inputs.

Motor actions can be organized into sequences, and animals use sensory feedback to adjust their choice of actions over time. While flies execute some grooming movements spontaneously, the anterior-to-posterior grooming progression is only observed in dust-covered flies [7, 8]. Here, we investigate how sensory stimulation induced by dust may contribute to the organization of the grooming sequence.

First, we systematically determine the function of each type of sensory organ in grooming. Previous work indicated the importance of mechanosensation for initiating the cleaning sequence, but the role of each type of mechanosensory organ was still unknown. We identify transgenic lines for genetically manipulating specific groups of sensory neurons. Though optogenetic activation of several types of sensory neurons can evoke grooming movements, sequential grooming is only induced by simultaneous activation of mechanosensory bristle neurons distributed over the body.

Various models have been offered to explain innate [7, 9] and learned [10–12] behavior sequences. In previous work, we proposed that the higher position of anterior cleaning in the grooming hierarchy can best be explained by a model based on parallel activation of different elementary grooming movements with a suppression hierarchy among them [7]. One implementation of this model suggested that the mutual suppression strength itself was asymmetric, with anterior behaviors more effectively inhibiting posterior ones. An alternative proposed that differences in activation strengths (sensory inputs to different body parts) could result in stronger induction of anterior behaviors. Though both mechanisms could coexist, here we provide evidence that unequal, spatially separated, competing sensory inputs are the key determinant of the initial choice of grooming movement.

During grooming, the sensory inputs induced by dust change over time as a result of dust removal, leading to a progression from anterior to posterior cleaning. We investigated how (1) the changing ratio of sensory inputs to different body parts or (2) the rate of removal of anterior dust (comparisons of dust levels on a given body part over time) affects grooming progression. Determining the ways sensory information contribute to behavior requires precise control of the animal’s sensory experience while simultaneously recording its behavior response. Because it is difficult to control the mechanosensory experience using naturalistic stimuli in free-moving animals, we adapted optogenetic [13] and fly-on-a-ball [14] systems to dissect mechanosensory integration. By manipulating optogenetic stimulation over time, we determine that anterior-to-posterior grooming progression results from the change in the ratio of anterior: posterior (A:P) sensory input over time and that the rate of change, as the anterior sensory stimulus declines, is not a critical factor.

## RESULTS

### Dust Induces Grooming Movements in an Anterior-to-Posterior Sequence

**Figure 1.**
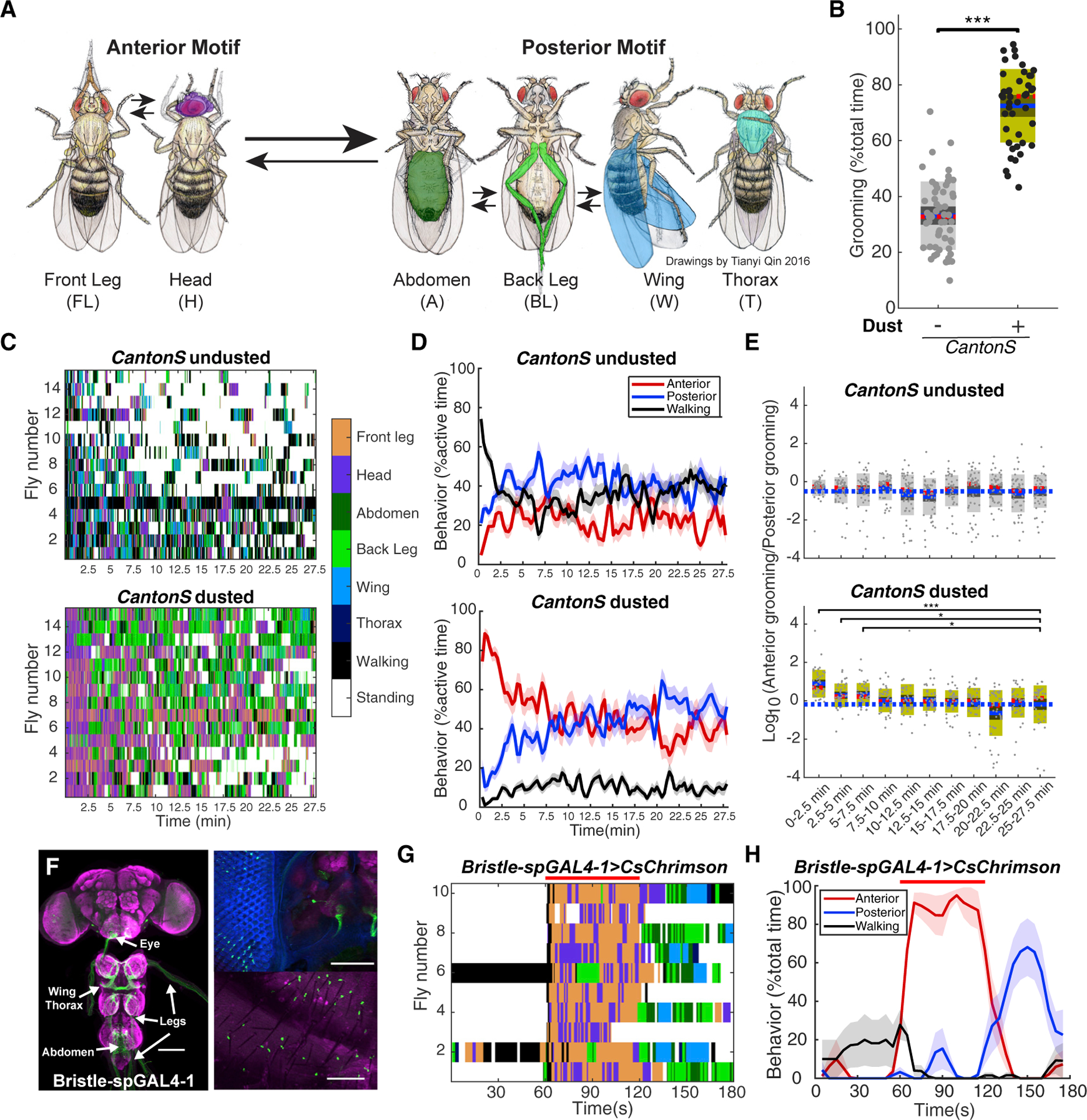
Dust or Optogenetic Activation of Mechanosensory Bristle Neurons Induces Anterior-to-Posterior Grooming Sequence (A) Diagram of stereotyped, recognizable grooming movements observed in *Drosophila melangaster*. Arrows indicate most common transitions, and the colored body parts correspond to the movements quantified in subsequent ethograms. Drawings by Tianyi Qin based on [16]. (B) The percent of time that undusted or dusted flies perform grooming behavior within 27.7 min total assay time (n ≥ 44). The mean is shown as a blue line; 95% confidence intervals for the mean are showed as dark shades. The median is shown as a dotted red line. One standard deviation is shown as light color shade. (C) Example ethograms of 15 individual *Canton S* flies in response to being shaken without or with dust generated by Automatic Behavior Recognition System (ABRS) classifier [15]. Each line is one individual. The color bar on the right stands for the color code used in the ethogram visualization. (D) Grooming progression for undusted or dusted *Canton S* flies. Behavior probabilities are calculated every 16 s in a sliding 32 s time window. Each data point is the average among all individuals (n ≥ 44). The shade stands for the standard error of mean. (E) The ratio of anterior to posterior grooming of undusted and dusted flies in 150 s time window. The dash blue line indicates the mean value at the last time window. (F) Expression pattern of Bristle-spGAL4–1 in central nervous system (CNS, left), eye (upper right), and abdomen (lower right). Green: anti-GFP. Magenta: anti-Bruchpilot in CNS, cuticle autofluorescence in abdomen. Eye facets are shown in blue. Scale bars, 100 mm.autofluorescence in abdomen. Eye facets are shown in blue. Scale bars, 100 μm. (G) Grooming response induced by optogenetic stimulation of bristle neurons. Optogenetic stimulation was given between 60 and 120 s, indicated by red line. Ethograms are color coded as in (C). (H) Grooming progression induced by optogenetic stimulation of bristle neurons. Behavior probabilities over total time are calculated every 2.5 s in a 5 s time window. See also Figures S1, S2, and S4. Wilcoxon rank-sum test (B) and Kruskal-Wallis test with Wilcoxon rank-sum post hoc (E) were used for significance tests. Asterisks represent the following p values: *p < 0.05, **p < 0.01, ***p < 0.001.

Our previous research showed that dust can induce an anterior-to-posterior grooming sequence in *Drosophila* [7]. We used a newly developed automatic behavior recognition system to analyze grooming in large scale [15]. Anterior and posterior grooming motifs contain different grooming subroutines. In the anterior motif, flies use their front prothoracic legs to clean their heads, and then discard dust through front leg rubbing. Posterior grooming motifs contain body sweeps with back metathoracic legs and back-leg rubbing (Figure 1A). Flies perform grooming, walking, and standing in our assay. Grooming was observed in undusted flies, and the leg movements are similar, but dust increases the grooming time (Figure 1B).

All dust-covered flies groom their anterior body parts first and then posterior ones, but the sequence is not exclusively unidirectional: flies switch back and forth between anterior and posterior grooming motifs and the behavior records from different individuals show variability (Figure 1C). We developed several ways to quantify grooming progression by aggregating data from many flies. One measures probability of performing anterior or posterior grooming movements or walking at each time point. Though anterior and posterior grooming probabilities are relatively stable throughout the assay in undusted flies, they vary reciprocally in dusted ones: the probability of anterior grooming starts high and declines, whereas the probability of posterior grooming starts low and increases over time. A “steady state” is eventually reached in which the probabilities of anterior and posterior grooming movements are approximately equal, although the probability of performing any grooming movement remains high (Figure 1D). We also quantified the ratio of anterior grooming to posterior grooming within 150 s intervals (abbreviated to A:P grooming ratio, referring to the behavioral outputs). In dusted flies, the A:P grooming ratio is highest at the beginning and decreases gradually. This trend is not observed in undusted flies, where the A:P grooming ratio is not significantly different among all intervals (Figure 1E).

Because dust can induce a sequence of grooming movements, we focus on two questions here: (1) what kind of sensory inputs are essential for grooming, and (2) how do these sensory inputs contribute to the behavioral sequence?

### Activation of Mechanosensory Bristle Neurons across the Whole Body Induces Anterior Grooming, Followed by Delayed Posterior Grooming

Mechanosensation is essential for insects’ sensorimotor control in complex environments [17]. Six types of mechanosensory organs are found in adult flies [4]. In previous experiments [18], acute activation of multiple kinds of mechanosensory neurons on both anterior and posterior body parts induced anterior grooming, which persisted briefly after the light stimulus terminated. Intriguingly, the flies then transitioned to posterior grooming, suggesting that they retained a memory of the previous whole-body stimulation and acted upon the posterior stimulation once suppression from the anterior behavior ended. But it was unclear which type of mechanosensory neuron plays the most essential role in this optogenetically induced sequence. Here, we systematically determine the function of each type in grooming.

Mechanosensory bristles are the most abundant mechanosensory exteroceptors distributed all over the body. Bristle deflection induced by contact, air puff, and parasites can induce targeted grooming [19–21]. We searched literature and image databases [22] to identify transgenic lines that target specific groups of sensory neurons and restrict expression further through split-Gal4 intersections [23]. We identified *Bristle-spGAL4–1*, which specifically labels approximately half of bristle neurons distributed over the body (Figure 1F). Activating bristle neurons with light for 1 min induced anterior grooming, whereas posterior grooming was observed immediately after light stimulus ended (Figures 1G, 1H, and S1C). A similar sequence was also induced by 5 s light activation (Figure S1N). The grooming bout structures–the way body sweeps and leg rubs alternate–induced by dust and bristle neurons activation are also very similar. Flies alternate between head sweeps and front leg rubs during the optogenetic stimulation and between body sweeps and back leg rubs after stimulus termination (Figure 1G). These results indicate that mechanosensory bristles may play an essential role in initiating the grooming sequence in dusted flies.

*Bristle-spGAL4–1* labels a few neurons in the central nervous system (CNS), but grooming can be induced with targeted light on legs, abdomen, or wings (which does not activate CNS neurons). This suggests the sequential grooming was induced by bristle neurons rather than neurons in the CNS.

### Activating Subgroups of Chordotonal Organs, Campaniform Sensilla, and Stretch Receptors Can Also Induce Grooming Movements but Not the Sequence

Other types of mechanosensory organs respond to different mechanosensory stimuli. Chordotonal organs can act as either exteroceptors or proprioceptors; they attach to cuticle or muscles through support cells. The Johnston’s organ (JO) is the largest group of chordotonal organs. It is located in the antenna, where it detects movements induced by sound, wind, or contact [24, 25]. In insect legs, the chordotonal organs encode leg position and movement [26]. Campaniform sensilla are associated with a cuticular dome and respond to deformation [27]. Hair plates are short, tightly packed sensory hairs, which are mainly located in leg joints [17]. Stretch receptors detect stretch between neighboring leg segments [28]. Multidendritic neurons can function as nociceptors in larva [29].

In principle, multiple types of mechanosensory organs may participate in grooming. Dust may be sensed by mechanosensory bristles to initiate grooming, whereas leg proprioceptors such as chordotonal organs and campaniform sensilla may provide position and pressure information required to target the legs accurately to specific body surfaces. It has been shown that grooming can be induced by optogenetic activation of antennal chordotonal organs and wing campaniform sensilla [18, 30]. Here, we extended our study to include all types of mechanosensory organs. Upon 1-min optogenetic activation through the red-light sensitive ion channel CsChrimson [31], grooming was induced by chordotonal organ neurons (CO-GAL4), campaniform sensilla neurons (Wing Haltere CS-spGAL4), and stretch receptor neurons (SR-GAL4). Activation of two other types of mechanosensory neurons, hair plate neurons (HP-GAL4) and multidendritic neurons (MD-GAL4), did not cause grooming (Figure S1B). One caveat is that some driver lines we used do not label all sensory neurons of that type, and neurons that are not labeled may still function in grooming.

However, the anterior-to-posterior grooming sequence was not induced by these three types of mechanosensory neurons: chordotonal organs induced head cleaning during light activation, but walking was observed immediately after light stimulus ended. Activating campaniform sensilla on wings and halteres together induced only wing grooming. Stretch receptors induced an equal amount of anterior and posterior grooming; the alternation between body sweeps and leg rubs was not observed (Figures S1C–S1M). These data suggest that mechanosensory bristles are key for the normal grooming progression.

We also identified transgenic lines that target chordotonal organs and campaniform sensilla neurons on specific body parts (Figure S2). Antennal chordotonal organ activation induced antennal grooming, but activating leg or abdominal chordotonal organs did not induce grooming (Figures S2A and S2J). Activating haltere campaniform sensilla alone induced grooming directed toward the halteres and back leg rubbing. Activating campaniform sensilla on legs did not induce grooming (Figures S2A and S2K). Therefore, the same type of mechanosensory organ on different body parts plays different roles in grooming.

### Sensory Neuron Inhibition Indicates that Multiple Mechanosensory Organs Participate in Dust-Induced Grooming

Gain-of-function experiments show that activation of these sensory neurons can induce grooming but do not demonstrate that these sensory neurons are the way flies normally sense dust. Loss-of-function experiments, in which flies are deprived of a sensory modality by genetic mutation or neuronal inactivation, would be ideal. Unfortunately, broadly inhibiting mechanosensory neurons usually causes lethality or extreme loss of coordination, masking specific grooming defects. To ameliorate this problem, we inhibited sensory neurons only on specific body parts.

**Figure 2.**
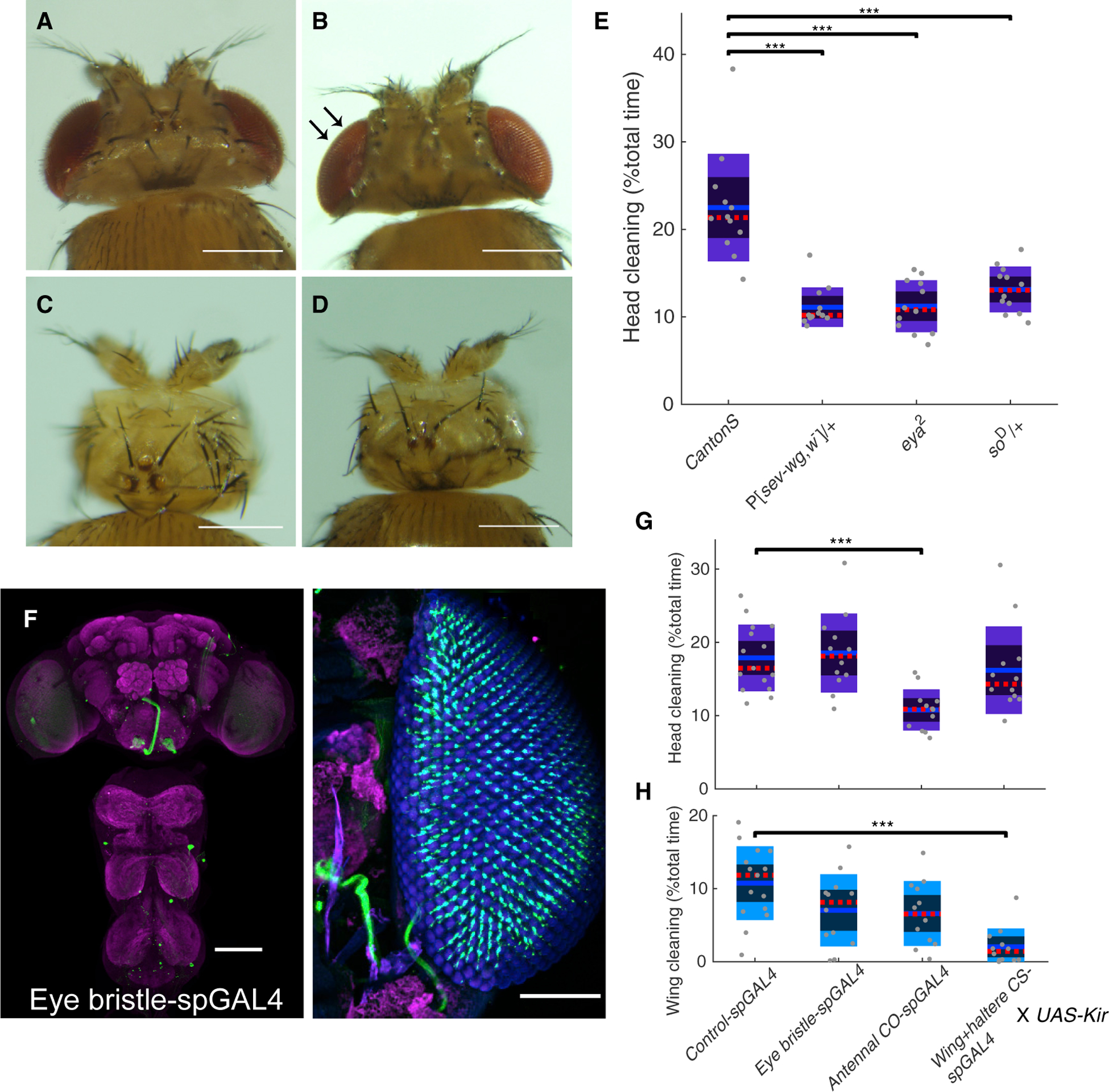
Multiple Types of Mechanosensory Organs Contribute to Dust-Induced Grooming (A–D) Interommatidial bristles are visible in wild-type *Canton S* eyes (A) but are absent in the P[*sev-wg*; *w*^−^] mutant, as indicated by arrows (B). The *eya*^*2*^ (C) and *so*^*D*^ (D) mutants lack eyes entirely. Scale bars, 250 μm. (E) These mutants show reduced head grooming, as indicated by the percent of time dusted flies spent in head grooming within 27.7 min (n = 12). (F) Expression pattern of *Eye bristle-spGAL4*. Green: anti-GFP. Magenta: anti-Bruchpilot. Eye facets are shown in blue. Scale bars, 100 μm. (G and H) (G) Inhibition of the neurons in the antennal chordotonal organs causes decrease in head cleaning, whereas inhibition of wing campaniform sensilla (H) causes decrease in wing cleaning compared to the amount of cleaning displayed by the control flies (n ≥ 12). Neurons were constitutively inactivated with *UAS-Kir2.1*. See also Figures S3 and S4. Kruskal–Wallis and post hoc Wilcoxon rank-sum test were used. Asterisks represent the following p values: *p < 0.05, **p < 0.01, ***p < 0.001.

Mechanosensory bristle neurons on the head or body may sense dust by bristle deformation. Even very small deflections can be detected by the mechanically gated ion channels located at the bristle base [32]. Alternatively, leg bristles might detect dust particles on other body parts during leg sweeps and rubs, either directly or as a difference in expected sweep force. Interommatidial bristles, located between each facet of the compound eye, are the most abundant bristle group, and their development can be disrupted by the P[*sev-wg*; *w*^−^] insertion (Figure 2B) [33]. Flies lacking eye bristles showed significantly reduced head grooming (Figure 2E). The whole compound eye can be eliminated by *so*^*D*^ or *eya*^*2*^ mutations (Figures 2C and 2D), and these eyeless flies also showed reduced head cleaning (Figure 2E). Because flies in the dark groom normally (Figures S4G and S4H), we attributed the reduced grooming phenotypes to loss of the interommatidial bristles. We also genetically silenced eye bristle neurons using a split-GAL4 driver line we identified (Figure 2F) to express Kir2.1, an inward-rectifying potassium channel [34], but these flies did not show significant changes in head grooming (Figure 2G). Neuronal inhibition through tetanus toxin (TNT) or GtACR1 also did not cause head grooming defects (data not shown). When the eye bristles are missing, much less dust accumulates on the head, but the normal amount of dust is still there when the bristles are present with neurons silenced. Because dust on compound eyes may be sensed by both eye bristles and front leg mechanosensory bristles–stimulated during head sweeps–this may explain why inhibiting eye bristles alone did not reduce head cleaning: the signals from legs compensate. Because inhibition of leg bristle neurons decreases basal walking activity and limb coordination [4], it is difficult to address their specific contribution to grooming.

We also tested whether other mechanosensory organs are important for dust sensing. Inhibiting antennal chordotonal organ neurons and wing campaniform sensilla neurons decreased grooming toward head and wings specifically (Figures 2G, 2H, and S3). These data demonstrate that multiple types of mechanosensory organs are involved in dust-induced grooming. Interestingly, inhibition of JO neurons by Kir caused a stronger phenotype than was seen in a previous study using TNT [30]. Because Kir inhibits neurons though membrane hyperpolarization, whereas TNT disrupts the release of vesicles at chemical synapses [35, 36], this difference suggests that the neurons in the JO may act through electrical synapses to promote grooming. Gap junctions have been observed between the JO and giant fiber [37]. Alternatively, JO neurons may just be less sensitive to TNT, like what occurs in mushroom body neurons [38].

Our data provided evidence that although multiple mechanosensory organs participate in dust sensation, mechanosensory bristles play the most important role in grooming sequence. The loss-of-function data do not contradict this view, but the strongest evidence supporting it is that their activation induces normal grooming movements in an anterior-to-posterior sequence.

### The Function of Taste, Vision, and Olfaction in Grooming Behavior

Stimulation of other sensory modalities, or disruption of expected stimulation, could also lead to grooming. We next investigated the function of gustatory, olfactory, and visual organs in grooming.

Gustatory sensilla are important taste organs in fruit flies [39]. We optogenetically activated gustatory neurons that sense sweet, bitter, or water with CsChrimson. Bitter taste neuron activation was reported to induce grooming [40], and we confirmed this finding (Figure S4A) but saw no anterior-to-posterior sequence (Figure S4B). Flies with a null mutation in a bitter receptor, Gr33a, did not show defects in grooming (Figure S4C), and inhibition of bitter taste neurons also did not cause grooming defects (Figure S4D). These data indicate that bitter taste is not necessary for dust-induced grooming. We tested different kinds of dust, from cornstarch to fungal spores, which presumably taste different, and observed sequential grooming (data not shown), further supporting that mechanosensory cues contribute more than taste to induce and organize grooming.

Grooming could help insects get rid of fungal spores attached to the cuticle. Fungi produce geosmin, sensed by the Or56a receptor, suggesting that olfaction could trigger grooming [41]. Antennal grooming may help insects maintain olfactory sensitivity [42]. Most conventional olfactory neurons are labeled by *orco-GAL4* driver line, but activating these neurons with CsChrimson did not induce grooming (Figure S4A). Two experimental manipulations were used to inhibit olfaction: an *orco* null mutant and amputation of the third antennal segment and maxillary palps, where all olfactory receptors are located. Both types of anosmic flies groomed normally in response to dust (Figures S4E and S4F), suggesting that olfaction is not essential.

*Drosophila* senses light with compound eyes and ocelli [43]. Flies could see dust on the eyes, or the dust could interfere with expected visual signals. We conducted grooming experiments in the dark, recording videos with infrared light, and observed normal grooming movements and hierarchy: flies still performed anterior cleaning first (Figures S4G and S4H). This supports the assertion that vision is not essential to dust-induced grooming behavior and does not explain why the eyes are cleaned first.

### Modeling Indicates that Unequally Distributed Mechanosensory Stimulation, Changing with Time, Can Account for Sequential Grooming

Our data show that mechanosensory bristle neurons induce grooming. We next investigated how the grooming sequence is shaped by sensory inputs. Two terms describe the anterior-to-posterior grooming sequence: “hierarchy” and “progression.” Hierarchy refers to which body part is groomed first or which is selected when there is competition. Progression represents the change in the choice of grooming actions over time.

**Figure 3.**
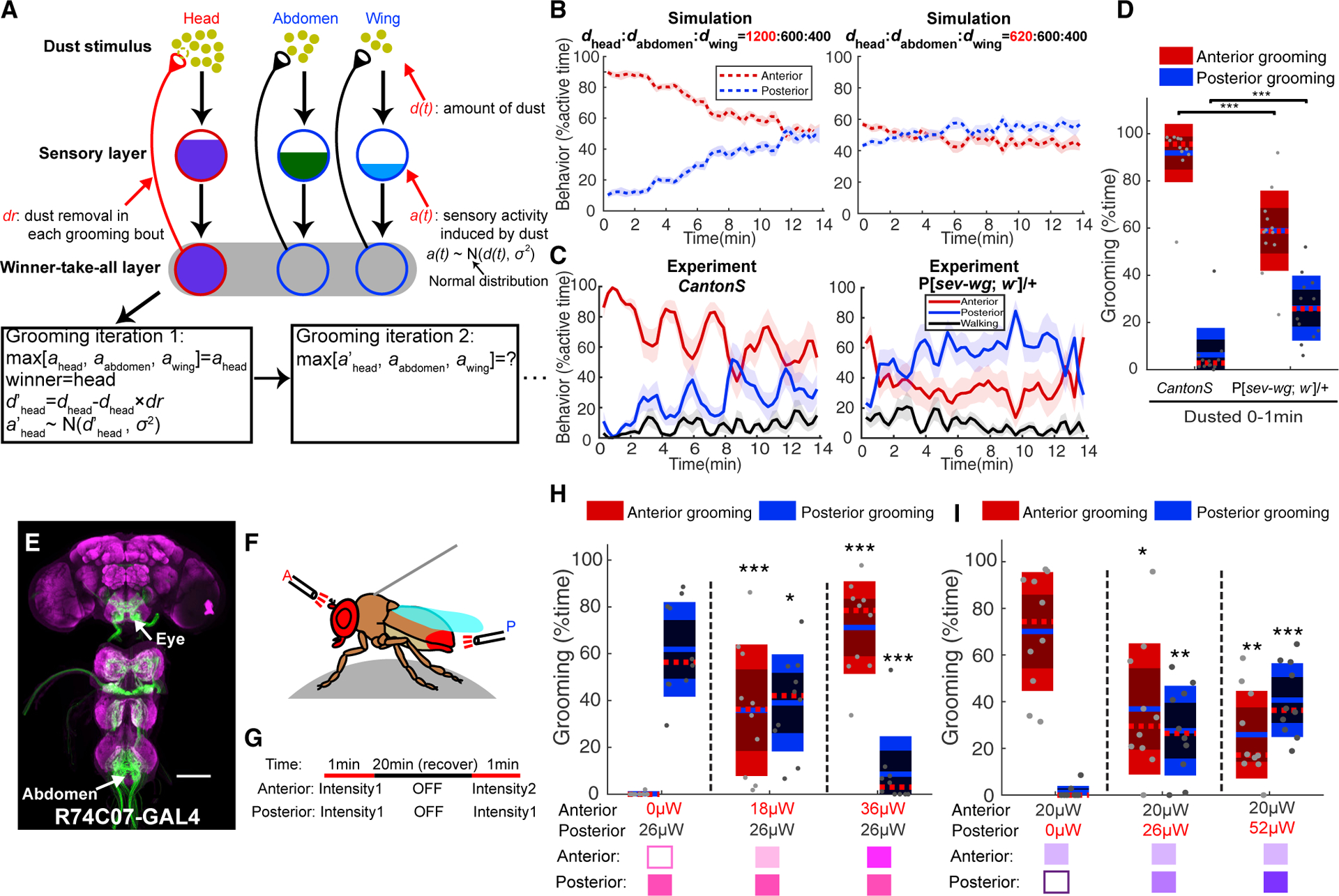
The Hierarchy of Grooming Movements Is Determined by Sensory Input Strengths to Different Body Parts (A) Schematic of grooming model with varied initial sensory inputs to different body parts (adapted from [7]). Dust (*d(t)*) activates sensory organs on different body part. Flies groom the body part with highest sensory activity (*a(t)*). The sensory activity term (*a(t)*), which represents a combination of amount of dust and number of sensory neurons, follows a normal distribution whose mean is *d(t)*. For each grooming iteration, the value of the term *d(t)* updates, as some percent of dust (*dr*) is removed from the body part that won the previous iteration. The change of dust distribution drives the sequential progression of grooming. (B) Model simulation with different initial dust levels. Left: Initial dust levels were set up according to bristle numbers on different body parts in wild-type files. Right: Simulation with decreased anterior sensory input reduced the initial ratio of anterior to posterior grooming. (C) Different grooming hierarchies were observed in dusted *Canton S* or P[*sev-wg*; *w*^−^]/+ flies that lack eye bristles (n = 12). Data are plotted as descripted in Figure 1D. (D) Quantification of the amount of anterior and posterior grooming during the first minute in dusted *Canton S* and P[*sev-wg*; *w*^−^]/+ shows that changing the number of eye bristles alters the initial amount of anterior grooming relative to posterior, lowering the anterior to posterior grooming ratio. (E) Expression pattern of *R74C07-GAL4* in eye bristle neurons that project to the subesphageal zone (SEZ), as well as posterior abdominal bristles that innervate the ventral nerve cord (VNC). Scale bars, 100 μm. (F) Schematic of “fly-on-a-ball” system. For optogenetic stimulation, two light fibers target anterior and posterior body parts separately. (G) Protocol used in optogenetic competition assay. Each fly was tested in two 1-min light stimulations. For each stimulation, the same posterior light (or anterior light) was coupled with different anterior light (or posterior light). 20 min recovery time was given between the two stimulations. (H and I) The change of grooming hierarchy as a result of varied sensory inputs (n = 10). (H) In tethered *R74C07 > ChrimsonR* flies, posterior light stimulus was kept constant while anterior light stimulus was increased in different experiments. An increased ratio of anterior to posterior grooming was observed. (I) When the anterior light stimulation level was held constant and the competing posterior light levels were increased, a decreased ratio of anterior to posterior grooming was observed. See also Figures S5 and S6. Wilcoxon rank-sum test was used for (D), (H), and (I). For (H) and (I), grooming time induced by each light condition was compared with posterior light only (H) or anterior light only (I) group. Asterisks represent the following p values: *p < 0.05, **p < 0.01, ***p < 0.001.

In our model, sensory organs all over the body are activated by dust simultaneously, but only one pair of legs can be used at a time. At each simulated grooming iteration, the body part that has the strongest sensory input is selected and cleaned. Because grooming removes dust, the drive to the selected body part is reduced, which may lead to a change of behavioral choice at the next evaluation. Our model has two layers: the sensory layer and the winner-take-all layer. The sensory layer quantifies dust-induced sensory input from different body parts. The winner-take-all layer compares sensory input strengths and selects one grooming subroutine for execution, thus converting probabilistic sensory inputs into a single behavioral output. Three variables are used in the sensory layer: *d(t)*, *a(t)*, and *dr*. *d(t)* represents the amount of dust on each body part. *a(t)* represents the sensory input induced by dust. *a(t)* follows a normal distribution whose mean is equal to current *d(t)*. *dr* indicates the dust removal rate, or the percentage of dust that is transferred from a body part to the legs in each grooming bout. It is defined as a percentage of the current *d(t)* rather than a constant amount to capture diminishing returns–less dust is removed when there is less on a body part. The initial value of *d(0)* and the constant value for *dr* are specified by the user, and first iteration selects the body part with the highest *a(0)* to be groomed. *d(t)* is re-calculated in each iteration, using *dr* applied to the currently selected body part to reduce *d(t)* and determine new values of *a(t)* for each body part. The winner-take-all layer then compares the updated sensory input level *a(t)* to select the next grooming action (Figure 3A). (Note that we did not model grooming bout durations here; these were drawn from the distribution obtained in experimental data.)

Sensory input levels should be a combination of the amount of dust and the number of bristles that detect it. To model the grooming sequence in wild-type flies, we assumed that each bristle gets the same amount of dust and set up initial dust distribution according to number of bristles on each body part: the head has ~1200, the abdomen has ~600, and the wings have ~400 [44–46]. This initial dust distribution reproduces the anterior grooming dominance observed in dusted wild-type flies. We also tested different values of *dr*. A simulation with *dr* = 0.2% generates a similar speed of grooming progression to what we observed in real flies. Anterior and posterior grooming probabilities became equal at the end of the simulation (Figure 3B), as they do when dusted flies reach steady state. Therefore, sequential grooming can be modeled by setting the initial sensory input strength to different body parts based on their number of mechanosensory bristles and then varying the subsequent drive based on targeted dust removal.

This model gives us guidance about how the grooming sequence can be affected by sensory inputs. With the help of model simulation, we next designed experiments to test how hierarchy and progression are affected by sensory inputs.

### The Anterior:Posterior Sensory Input Ratio Dictates Grooming Hierarchy

The grooming hierarchy can be observed by the A:P grooming ratio–the relative amounts of anterior and posterior grooming as described in Figure 1E. In our simulation, this ratio is affected by sensory input strength, and so reducing initial dust values for the head led to decreased anterior grooming (Figure 3B). The predictions of the model led us to devise additional experimental tests. It has been challenging to apply specific amounts of actual dust to fly body parts, but there are several alternatives. First, we lowered sensory input to the eye by applying dust to mutant flies lacking eye bristles. Both the amount of dust retained on the eyes and the sensory neurons that detect it were reduced. This resulted in reduced initial A:P grooming ratio (Figures 3C and 3D) and supports our prediction that sensory input strengths establish anterior dominance in the hierarchy.

Optogenetic experiments with light aimed at specific body parts allow us to control sensory input strength with more accuracy. We expressed ChrimsonR [31] in mechanosensory bristle neurons, tethered the fly, and then used two independently targeted light sources to separately activate anterior and posterior body parts (Figure 3F). *R74C07-GAL4* labels mechanosensory bristle neurons on eyes and posterior abdomen (Figure 3E), providing better separation of the activation zones. We gave the same fly two different 1-min light activation protocols and compared grooming behaviors induced by different light conditions (Figure 3G). In the first set of experiments (Figure 3H), for each pair of light presentations, we held the posterior light intensity constant and varied the level of anterior stimulation. The posterior activation is sufficient to induce posterior grooming in the absence of competition, but higher levels of anterior activation drove an increase in anterior grooming at the expense of posterior grooming. In the second set of experiments, the same anterior illumination level was paired with different posterior stimulations. High posterior light levels were enough to swing the balance toward posterior grooming (Figure 3I). Similar results occur with sensory competition between head and wings (Figures S5A and S5B). Previous studies showed that animals perform input comparison between left and right sensory organs [47, 48]. Our model simulation suggested that spatial sensory comparison between anterior and posterior body parts is also an essential part of behavior choice. By using classical genetic mutants and a novel optogenetic assay, we experimentally demonstrated this prediction: the initial grooming hierarchy is determined by the ratio of sensory input strength to different body parts.

### Grooming Progression Is Absent during Constant Sensory Stimulation

**Figure 4.**
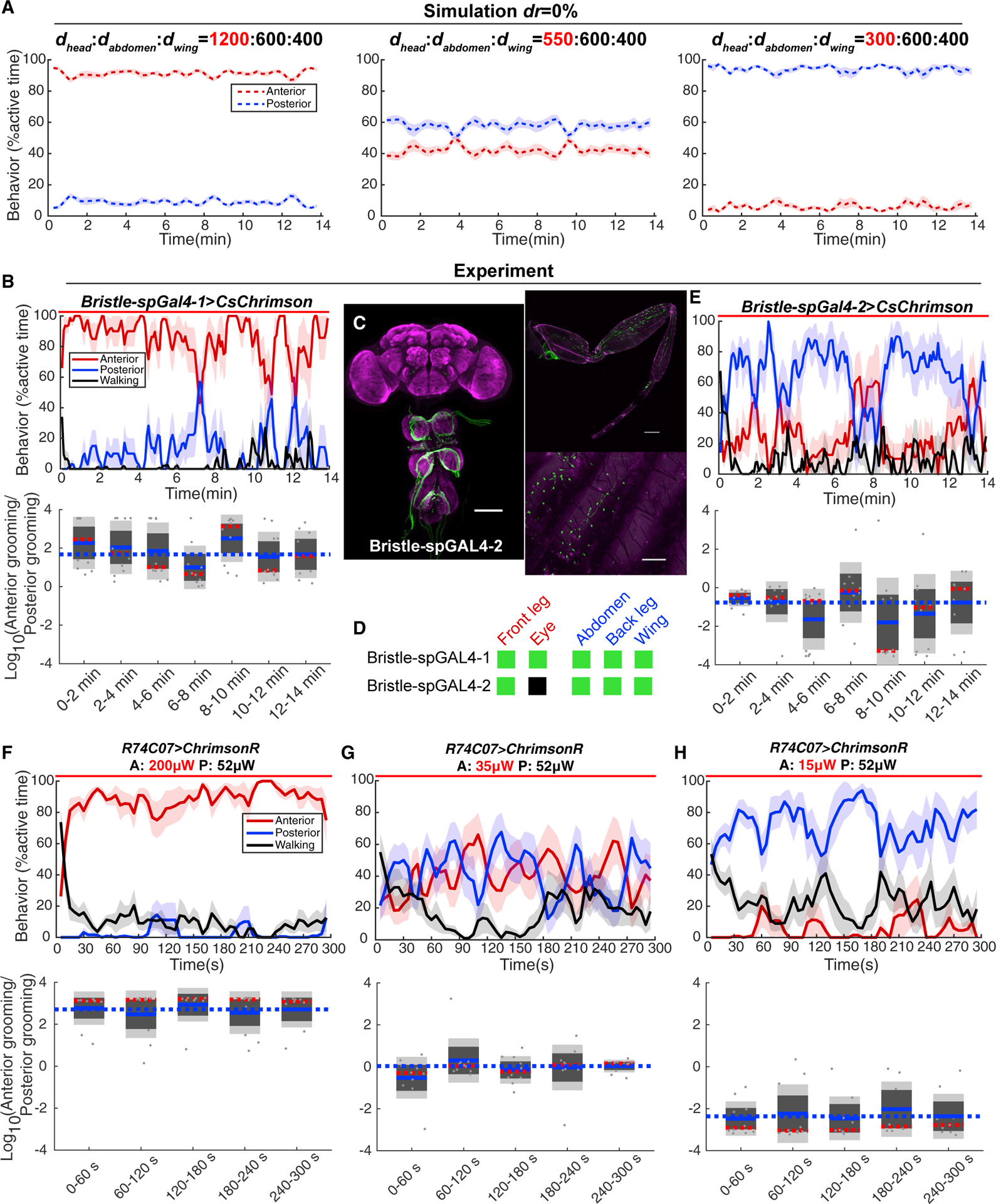
Grooming Progression Requires Changing Sensory Stimulus (A) Model simulation with constant dust levels over time (*dr* = 0%). Data are plotted as descripted in Figure 1D. (B) Using spGAL4 lines to restrict expression of CsChrimson to mechanosensory bristle neurons on different body parts and applying light from below to freely moving flies (n = 10). The probability of anterior grooming (red), posterior grooming (blue), or walking (black) is calculated every 5 s in a 10 s time window. Below, the ratio between anterior and posterior grooming is calculated in 2-minute time windows. The blue dashed line indicates the mean value for the last time window. No significant difference was found between each time window (Kruskal–Wallis test). (C) Expression pattern of *Bristle-spGAL4–2* visualized with *UAS-mCD8-GFP* in CNS, leg, and abdomen. Scale bars, 100 μm. (D) Summary table of expression patterns of two bristle neurons spGAL4 lines. Green indicates expression, and black indicates no expression. The detail expression pattern of *Bristle-spGAL4–1* can be found in Figure 1F. (E) Same as (B), using Bristle-spGAL4–2. **</p>**(F–H) In tethered *R74C07 > ChrimsonR* flies, constant level anterior and posterior light stimulus was given for 5 min (n = 10). In (F), anterior illumination strength is 200mW. In (G), anterior illumination is 35mW, and in (H), it is 15mW. Behavior probabilities and anterior to posterior grooming ratio is quantified as in (B). See also Figures S5 and S6.

Flies remove dust particles from specific body parts during grooming. In our model, we simulated this by including the term *dr*. This removal, and the corresponding change in the distribution of sensory inputs, is critical for the progression of grooming action choice: when we set dust removal to zero in the simulation, there is no anterior-to-posterior grooming progression (Figure 4A). The probabilities of anterior and posterior grooming stayed constant and corresponded to their initial activation levels (*a*): when the A:P sensory input ratio was high, anterior grooming always dominated, and when the A:P sensory input ratio was low, posterior behaviors dominated over the whole time course.

We then used both genetic reagents and mechanosensory competition experiments to test this prediction. First, we gave bristle neurons distributed over whole body,*Bristle-spGAL4–1*, constant optogenetic stimulus for 14 min (in undusted flies). The A:P grooming ratio stayed similar over time (Figure 4B). We identified *Bristle-spGAL4–2* that targets bristle neurons on the body and legs but not eye bristle neurons (Figures 4C and 4D). Activating these neurons mainly induced posterior grooming (Figure 4E). Regardless of the starting stimulation ratio, under constant illumination, no obvious grooming progression was observed. The fly-on-a-ball setup gave us more freedom to separately control the sensory inputs to different body parts. Using the *R74C07-GAL4* line, we tested 5-min constant light stimulus in three conditions: high anterior light intensity, similar anterior and posterior light intensity, and high posterior light intensity. The ratio of A:P grooming behavior was determined by the initial A:P sensory input ratio and stayed constant over time in all three conditions (Figures 4F–4H). These results confirmed our previous conclusion that the grooming hierarchy is determined by the ratio of sensory input strengths from different body parts and demonstrated that the change of sensory stimulation over time is necessary for grooming progression. But what aspect of the dynamics are the flies measuring to determine which body part to groom as time goes on?

### The Timing of Grooming Progression Depends on Changing Sensory Inputs

**Figure 5.**
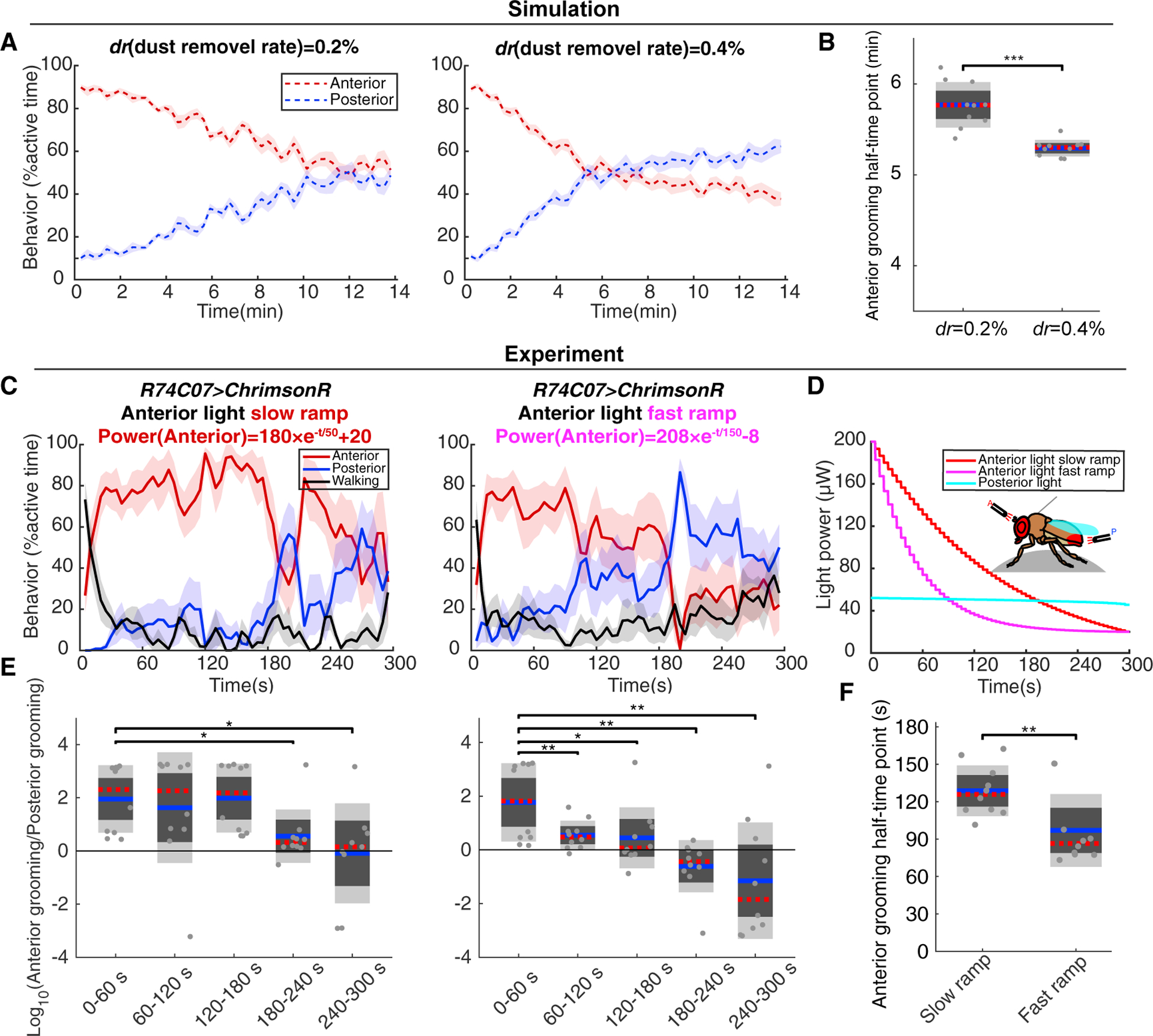
The Timing of Grooming Progression Depends on Changing Sensory Inputs (A) Model simulation with different *dr* shows that the transition to higher probability of posterior grooming occurs earlier when the *dr* is higher, (as in Figure 3B, *d*_*head*_ = 1200, *d*_*abdomen*_ = 600, *d*_*wing*_ = 400; each simulation was performed 10 times). Data are plotted as described in Figure 1D. This is also quantified in (B) as the time points at which simulation flies finish half of their total anterior grooming. (C–F) Tethered *R74C07 > ChrimsonR* flies were tested in two different light ramps. In different experiments, the same light ramp was given to posterior body parts, whereas a slow or fast light ramp was given to the anterior body parts (n = 10). (C) Behavior probabilities are quantified as in Figure 4B. (D) Light conditions used in the experiments. (E) Quantification of ratio between anterior and posterior grooming in each 60 s time window. (F) The anterior grooming half-time points under different light conditions. See also Figure S5. Kruskal-Wallis test with Wilcoxon signed-rank test post hoc were used for significance tests. Asterisks represent the following p values: *p < 0.05, **p < 0.01, ***p < 0.001.

To investigate how the change of sensory inputs affects grooming progression, we performed simulations with different *dr* values. Increasing the rate of dust removal shifted the time at which the posterior grooming percentage overtakes anterior earlier, indicating faster progression (Figure 5A). The time point when a fly has finished half of the total anterior grooming it will do is also a measure of the grooming progression speed (Figure S5D). Flies with larger dust removal values progress to posterior grooming faster, resulting in earlier anterior “half-times” (Figure 5B).

We used the fly-on-a-ball system to test predictions from simulation. Targeting light to anterior and posterior body parts allows us to control the relative sensory inputs and vary their intensity over time. We tethered R74C07 *> ChrimsonR* flies and applied a very gradually decreasing posterior light stimulation selected to be sufficient to induce posterior grooming in the absence of competing stimuli (Figure 5D). We coupled this posterior stimulation with two different anterior light intensity ramps. When the anterior light levels decreased slowly at the beginning and fell under posterior light levels late (red, slow ramp), flies reached the anterior and posterior grooming equilibrium point at ~270 s and achieved half-time around 120 s. When the anterior light levels decreased faster at the beginning and fell under posterior light levels earlier (purple, fast ramp), flies transitioned to predominantly posterior grooming sooner (180 s) and achieved half-time at 90 s (Figures 5C and 5F). An alternative way to quantify the grooming progression is to examine the A:P grooming ratio in sequential 60 s time bins. The A:P grooming ratios shift significantly at 60 s with a faster anterior ramp but only after 180 s with the more gradual one (Figure 5E). The faster decrease in anterior stimulation levels mirrors the higher dust removal values in the simulation (Figure 5A, right panel) and may mimic more efficient dust removal in dirty flies. Therefore, the way the relative sensory inputs change influences the timing of grooming progression.

### Iterative Spatial Comparisons of Mechanosensory Inputs, rather than Temporal Comparisons Showing Anterior Dust Removal Rate, Is Key for Grooming Sequence

**Figure 6.**
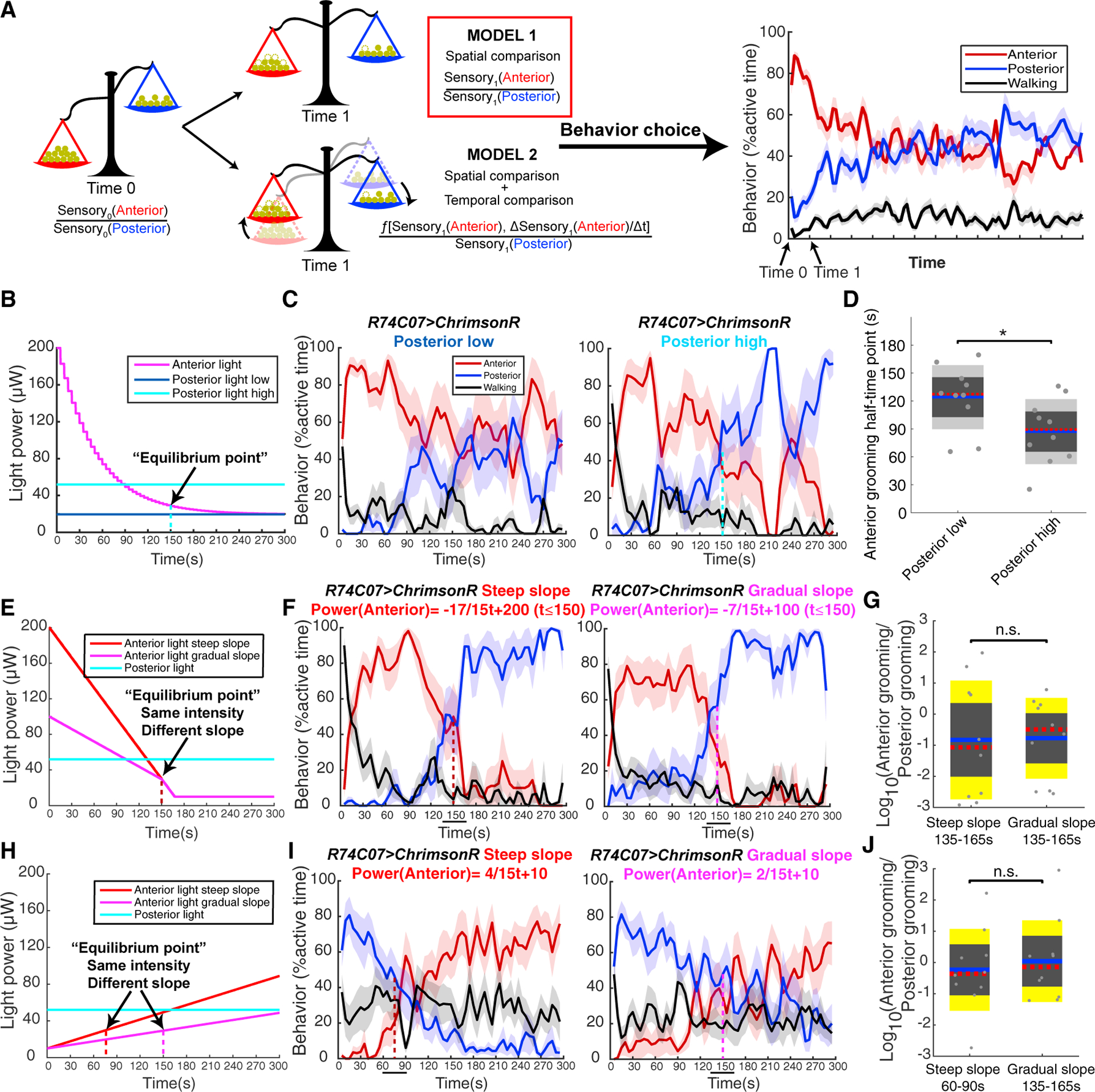
The Changing Ratio of Sensory Input Strengths to Different Body Parts, rather than the Rate of Change of Anterior Sensory Input, Is Key for the Progression of Grooming (A) Two different models can explain how the change of sensory inputs drives the change of behavior choice. In the first model, only the ratio of sensory input strengths to different body parts determines the behavior choice at that time point. In the second model, both the sensory input ratios and the temporal change of sensory information are important. (B–D) Tethered *R74C07 > ChrimsonR* flies were tested in two light conditions. In each condition, different level of posterior light was coupled with same anterior light curve (n = 10). The equilibrium point (30μW:52μW) in posterior high condition where the probability of anterior grooming equals to posterior grooming is shown by a light blue dashed line. (B) Change of light power over time in each experiment condition. (C) Behavior probabilities at different time points is quantified as in Figure 4B. (D) The anterior grooming half-time points under different light conditions. (E–J) In different experiments, same constant light was given to posterior body part; anterior light crossed the same equilibrium point (30μW:52μW, indicated by arrow) at different slopes (n = 10). Vertical dashed light indicates the position of “equilibrium point.” (E and H) Change of light power over time in each experiment condition. (F and I) Behavior probabilities at different time points is quantified as in Figure 4B. (G and J) The ratio of anterior grooming to posterior grooming within the 30 s time windows around the target light intensity point. The time windows are indicated by black solid lines in (F) and (I). See also Figures S5 and S6. Wilcoxon signed-rank test was used for significance tests. Asterisks represent the following p values: *p < 0.05, **p < 0.01, ***p < 0.001.

Two possible mechanisms can explain the result that faster anterior light decrease leads to faster progression. (1) Faster sensory input change reduces the A:P sensory input ratio faster. Flies may frequently compare the levels of sensory input to anterior versus posterior body and switch when they become close to equal. Animals respond to the absolute level of sensory inputs, but they also monitor how these sensory inputs change over time [5, 6, 49]. (2) Alternatively, flies may measure the temporal change of sensory input to a specific body part. Faster anterior sensory input change indicates more efficient anterior grooming, which may drive the grooming progression to posterior body parts (Figure 6A). We designed optogenetic competition experiments to investigate which spatial and temporal comparisons contribute to the grooming sequence and thus discriminate between these possible mechanisms.

As shown above, initial grooming movement choice is determined by initial A:P sensory input ratio. We further tested whether flies perform iterative spatial comparisons throughout the whole grooming sequence. We applied the same anterior stimulation (decreasing with an exponential function) in competition with either low (dark blue) or high (light blue) posterior stimulation levels (Figure 6B). In both light conditions, anterior grooming dominated initially, given flies almost exclusively performed anterior grooming during the first 50 s (Figure 6C). If the amount of anterior stimulation relative to posterior stimulation (A:P input “ratio”) is important, then flies in the high-posterior case should transition first. Alternatively, if the rate at which anterior stimulation decreases (temporal comparison) is the only key criteria for transition, the flies should show the same grooming progression in both experiments since the same anterior light protocol was used. The former was what we saw, indicating that “ratio” is important. In low-posterior case, anterior grooming dominated over posterior grooming for the whole 300 s. In high-posterior case, posterior grooming successfully out-competed anterior grooming at 30μW:52μW, which we call the “equilibrium point” (Figure 6C). *R74C07-GAL4* labels more eye bristle neurons compared with abdominal bristle neurons, which may explain why lower anterior light intensity compared with posterior light is required to reach equilibrium. The different half-times of 90 s versus 125 s (Figure 6D) also indicate that faster progression was induced by high posterior light stimulus. Therefore, flies not only compare the initial sensory input to different body parts, but also make iterative spatial comparisons throughout grooming. This iterative spatial comparison is essential for the change of behavior choice.

Next, we used this equilibrium point (30μW:52μW) (Figures 6B and 6C) to test whether the behavior choice can also be affected by the temporal comparison of sensory inputs to the anterior region. We gave flies constant posterior light intensity and presented different anterior stimulus protocols that ramped through the equilibrium point (indicated by arrow) at different slopes (Figure 6E). Interestingly, the transition times from majority anterior grooming to majority posterior grooming happened at almost the same time (150 s) in both conditions (Figure 6F). An alternative measure of behavior choice, the A:P grooming ratio around that point, also showed no significant difference (Figure 6G). This indicates that this temporal comparison–the rate of change of anterior mechanosensory input–is not critical for the grooming progression.

The anterior-to-posterior grooming transition occurs when the sensory input ratio reaches a certain threshold–but does the history matter at all? We tested whether reversing the ramp of anterior stimulation would alter the transition point, starting with low anterior illumination and increasing it to approach the equilibrium point (30μW:52μW; two black arrows) from below at different time points, using various slopes (Figure 6H). We found that A:P grooming ratios were similar at that target point, regardless of how it was approached (Figures 6I and 6J). Thus, we demonstrate that the slope value and sign do not affect grooming movement choice but that the current ratio of anterior-to-posterior sensory input, which changes over time, is the essential determinant. These results were confirmed with independent reagents and light conditions (Figure S6). We conclude that iterative instantaneous spatial comparisons between sensory inputs to different body parts drive changing grooming movement choice over time, leading to an anterior-to-posterior grooming sequence in dusted flies.

## DISCUSSION

The neural mechanisms for processing sensory signals and the way this information is used to select among behaviors remain open questions. Evaluating which kinds of sensory inputs can initiate a behavior is the first step in understanding this process. In this work, we systematically investigated the role of different types of sensory organs in *Drosophila* grooming. We found that multiple types of mechanosensory organs are involved in grooming, but mechanosensory bristles are most essential for grooming sequence: their activation induces the anterior-to-poster grooming progression and cyclic switching between body cleaning and leg rubbing. Electrophysiology recordings have shown that a mechanosensory bristle can respond to displacements as small as 100nm [32]. Therefore, mechanosensory bristles could detect small deflections induced by dust particles. JO also participates in dust sensing. JO C/E neurons can respond to movements as small as a few micrometers [24, 25]. Thus, JO neurons could be activated by antenna displacements induced by dust weight. Parasites, mechanical irritants, and damage will cause changes in the position and mechanical load of body parts, which may be sensed by stretch receptors and campaniform sensilla. Grooming can help *Drosophila* remove debris, increase sensory acuity, and restore proper position of body parts, so it is an appropriate response to mechanosensory stimulation. Future work will be required to determine the exact mechanism of dust sensing by different mechanosensory organs.

Behavioral analyses suggest challenges the nervous system solves. For grooming, the presence of a somatotopic map can be inferred because of the precision with which the legs move to sweep stimulated bristles [19]. Some ability to ignore self-generated sensory stimulation also seems likely, because flies do not get stuck in constant grooming loops triggered by bristle deflections during their own leg sweeps. Interhemispheric neurons may coordinate in-phase and out-of-phase leg movements for symmetric body sweeps and asymmetric leg rubs. Intersegmental neurons mediate mutual exclusivity between front and back leg movements to maintain posture and balance.

The CNS integrates information from different sensory modalities and body parts. Our experiments show that during grooming, flies frequently compare sensory input strengths from anterior and posterior body parts to choose grooming actions. Mechanosensory bristle and proprioceptive neurons in the leg extend axons into distinct areas of the leg neuropils of the ventral nerve cord. Bristle neurons from the body also project to the ventral nerve cord and abdominal bristle neurons arborize in the abdominal ganglia [4], whereas interommatidial bristle neurons and head bristle neurons extend primarily into the subesophageal zone of the brain. Sensory and motor neurons have been characterized, and some neurons that receive sensory neuron inputs from the left and right legs have recently been identified [50], but the majority of interneurons that compare sensory inputs from different body parts remain to be found.

Modeling guided experimental tests using our optogenetic competition assay. We determined which spatial and temporal comparisons matter for behavior choice. For grooming, we now know that comparisons between mechanosensory bristle neurons on anterior and posterior body parts are critical. Using a combination of anatomically guided selection of genetic reagents and behavioral screening, we previously mapped much of the neural circuitry controlling antennal grooming [30]. Our future work will employ this approach to identify the neural circuits that control posterior grooming behaviors and mediate the selection among anterior and posterior cleaning routines. Sensory integration and action selection are common challenges animal brains must solve to coordinate effective behaviors. Demonstrating the behaviorally relevant comparisons is the first step to mapping the circuit motifs that accomplish them.

## STAR★METHODS

### LEAD CONTACT AND MATERIALS AVAILABILITY

Further information and requests for resources and reagents should be directed to and will be fulfilled by the Lead Contact, Julie H. Simpson (jhsimpson@ucsb.edu). This study did not generate new unique reagents.

### EXPERIMENTAL MODEL AND SUBJECT DETAILS

Flies *Drosophila melanogaster* were reared on common cornmeal food in 25°C incubators on a 12 hr light/dark cycle. For optogenetic experiments, larvae were raised on normal food. After eclosion, 1-day old adults were transferred into food containing 0.4 mM all-trans-retinal and reared in the dark for another two days. For olfactory organs amputation, antennae and maxillary palps of 3-day *Canton S* males were removed by fine tweezers. They were given three days to recover before dusting experiments. Eye bristle and compound eye mutants were backcrossed with *Canton S* for five generations before grooming experiments. A full list of fly lines can be found in the Key Resources Table.

### METHOD DETAILS

#### Identification of fly lines that target sensory neurons

We performed literature research to identify transgenic lines that target different groups of sensory neurons. To identify additional driver lines, we performed a visual screen on CNS expression patterns in the Flylight database [43]. Candidate driver lines were crossed with GFP effector line, GFP expression in sensory neurons was confirmed by peripheral nervous system (PNS) imaging. Split Gal4 approach [22] was used to further refine the expression to sensory neurons.

#### Immunofluorescence and confocal imaging

For CNS immunostaining, whole flies immobilized with insect pin on abdomen were fixed in 4% PFA for 2 hours on nutator at room temperature. After three 1 min wash in PBT, flies were dissected in PBS buffer to get the whole CNS. CNS samples were further washed by three times in 1 min PBT and then blocked for 30 min in 4% NGS. Staining with primary antibody was performed in 4°C overnight on nutator. Samples were then washed 3 times for 20 min in PBT. Secondary antibody incubation was performed for 2 hours in room temperature. Samples were washed again in PBT for 3 times; mounted in VectaShield for imaging. PNS dissection and eye bristles immunostaining was performed using the published protocol [10]. In short, whole flies were washed in 100% ethanol and then PBS, specific body parts were then pulled and mounted in VectaShield on microscope slides for imaging. The following primary antibodies were used: chicken polyclonal to GFP (Abcam 13970, 1:500) and mouse monoclonal brp antibody (DSHB nc82, 1:200). The secondary antibodies were anti-chicken Alexa Fluor 488 (Invitrogen Molecular Probes A-11039, 1:500) and anti-mouse Alexa Fluor 633 (Invitrogen Molecular Probes A-21052, 1:500). Confocal images were taken on a Zeiss LSM710 microscope. Images were then processed in ImageJ.

#### Morphology of eye bristle mutants

Eye photos of male flies were taken through an SZX 12 Olympus stereomicroscope at different Z positions. Z series for each fly were registered through BUnwarpJ (https://imagej.net/BUnwarpJ) and converted into single image through Extended Depth of Field plugin (http://bigwww.epfl.ch/demo/edf/).

#### Recording and analysis of dust-induced grooming

Three chambers were used in fly dusting assay: dusting chamber (24 well corning tissue culture plate #3524), transfer chamber and recording chamber. Dust-induced grooming assays were performed in 21–23°C. 4–7 days male flies were anesthetized on ice and transferred to the middle four wells of transfer chamber. 10-day old males were used in Kir inhibition experiments to increase the expression level of Kir. Flies were left in transfer chamber for 15 min to recover. Around 5 mg Reactive Yellow 86 dust was added into each of the 4 middle wells of dusting chamber. Before use, dust was baked in a 160°C oven overnight to remove extra moisture. For fly dusting, transfer chamber was aligned with dusting chamber. Flies were tapped into dusting chamber and shaken for 10 times. After dusting, flies and dust were transferred back into transfer chamber. Transfer chamber was banged against an empty pipette tip box to remove extra dust. Dusted flies were then immediately tapped into recording chamber for video recording. The whole dusting process was performed in a WS-6 downflow hood. As undusted control, flies with the same genotype were shaken in chambers without dust. At least 10 individuals were recorded for each genotype.

30 Hz videos were recorded for 50,000 frames (27.78 min) with a DALSA Falcon2 color 4M camera. A white LED ring right was used for illumination. Infrared backlight was used for grooming experiments in the dark. Videos were processed through ABRS to generate ethograms. Grooming modules were described previously [7].

#### Optogenetics experiments of free-moving flies

After cold anesthesia, flies were left to recover in recording chamber for at least 20 min. Custom-made LED panels (LXM2-PD01–0050, 625nm) were used for light activation from below. 20 Hz 20% light duty cycle was used in all experiments. LED power was adjusted according to the expression level and behavioral response of different lines. Light intensity was measured by Thorlabs S130VC power sensor coupled with PM100D console. The light intensity used in the experiments are: *Control-spGAL4* (8.4 mW/cm^2^), *Bristle-spGAL4–1* (0.84 mW/cm^2^), *Bristle-spGAL4–2* (0.84 mW/cm^2^), *Wing+haltere CS-spGAL4* (5.6 mW/cm^2^), *Control-GAL4* (8.4 mW/cm^2^), *CO-GAL4* (1.4 mW/cm^2^), *SR-GAL4* (8.4 mW/cm^2^), *HP-GAL4* (5.6 mW/cm^2^), *MD-GAL4* (5.6 mW/cm^2^), *Antennal CO-spGAL4* (5.6 mW/cm^2^), *R21D12-GAL4* (8.4 mW/cm^2^), *R73D10-GAL4* (5.6 mW/cm^2^), *R86D09-GAL4* (5.6 mW/cm^2^), *VT028607-GAL4* (8.4 mW/cm^2^), *R14F05-GAL4* (8.4 mW/cm^2^), *Gr33a-GAL4* (5.6 mW/cm^2^), *Gr64f-GAL4* (5.6 mW/cm^2^), *Ppk28-GAL4* (5.6 mW/cm^2^), *Orco-GAL4* (5.6 mW/cm^2^), *Or56a-GAL4* (5.6 mW/cm^2^), *Control-LexA* (5.6 mW/cm^2^), *R42G12-LexA* (5.6 mW/cm^2^). 30Hz videos were recorded by IDS UI-3370CP-C-HQ camera and manually annotated in VCode or automatically annotated by ABRS (https://github.com/AutomaticBehaviorRecognitionSystem/ABRS).

#### Fly-on-a-ball experiment

Experimental rig was set up as protocol described previously [40, 44] with modifications. In short, 3 days female was tethered to a size 1 insect pin through UV glue. Air flow was used to support the 10mm diameter foam ball (LAST-A-FOAM FR-7120 material). Air flow (500–600 mL/min) passed through water before foam ball for humidification. Two Doric Lenses fiber LEDs (CLED_635) with custom-made collimator were used to target head and posterior end of abdomen. Thorlabs NE513B neutral density filters were used to adjust light intensity. To determine the light intensity, we first did preliminary experiments to see which light combination give us approximately equal amount of anterior and posterior grooming. Then we used that intensity with changed anterior light intensity or changed posterior light intensity to investigate how sensory input ratio change affects behavior choice. Because it is hard to measure the illumination area, LED light power rather than intensity was used. LED driver was connected with National Instruments USB-6008 DAQ to control light ramp. For *R52A06 > ChrimsonR* flies, 20 Hz 20% light duty cycle was used for anterior light stimulation, 20 Hz 50% light duty cycle was used for posterior light stimulation. 20 Hz 20% light duty cycle was used for both anterior and posterior light stimulations in *R74C07 > ChrimsonR* flies. Each fly was tested in two different light conditions. The order of light conditions was random. 20 min recovery time was given between different conditions. 30Hz videos were recorded with a Point Grey BFS-U3–13Y3M-C camera and manually annotated in VCode.

#### Computational model

*d(t)* stands for dust amount on different body parts. For simulation of *Canton S* flies, *d(t)* was set up according to mechanosensory bristle numbers on different body parts. Initial dust on front legs and back legs was set to be 200. *a(t)* represents neural activities induced by dust. It follows a normal distribution whose mean is *d(t)*, the relationship between *d(t)* and s*(t)* is estimated according to the bristle electrophysiology recordings [32]:

$$a(t)\sim \text{N}\left(d(t),\sigma {\left(t\right)}^{2}\right),\sigma (t)=d(t)/5$$

Winner-take-all layer determines the body part which has the highest neural activity (*a*_*body part*_*(t)*) as the winner. The winner body part will be groomed in this grooming iteration. If the winner is leg, some percent of dust (10*dr*) will be discarded. Otherwise, some percent of dust (*dr*) will be transformed from winner body part to the corresponding legs:

dfront/back(t=1)legs=dfront/back(t)legs−dfront/back(t)legs*10dr(winnerisleg)

dwinner(t=1)=dwinner(t)−dwinner(t)*dr,dfront/back(t+1)legs=dfront/back(t)legs+dwinner(t)*dr(winner is other body parts)

We did not model the grooming bout duration. It was drawn from duration distributions of different grooming modules we got from two manually labeled dusted *Canton S* ethograms.

### QUANTIFICATION AND STATISTICAL ANALYSIS

Data analysis was performed in MATLAB 2016b and 2017b. Wilcoxon signed-rank test was used for two related samples. Wilcoxon rank-sum test was used for two independent samples. Kruskal-Wallis test with Wilcoxon rank-sum post hoc were used for three or more independent samples.

Data was plotted with notBoxPlot (https://github.com/raacampbell/notBoxPlot) function. Each dot is one fly. The mean is shown as a blue line, 95% confidence intervals for the mean are showed as dark shades. The median is shown as a dotted red line. 1 standard deviation is shown as light color shade.

shadedErrorBar (https://github.com/raacampbell/shadedErrorBar) function was used for grooming progression figures. For dusting experiments or model simulations. Behavior probabilities were calculated every 16 s in a sliding 32 s time window. For optogenetic experiments with 5 s and 1min light activation, behavior probabilities were calculated every 2.5 s in a sliding 5 s time window. For optogenetic experiments with 5min and 14min light activation, behavior probabilities were calculated every 5 s in a sliding 10 s time window. Each data point is the average among all individuals. The shade stands for the standard error of mean.

To quantify the ratio of anterior grooming to posterior grooming in each time window, we first calculated the duration (as frame number) fly performed anterior or posterior grooming for that interval. If the fly did not perform any grooming behavior during that period. That time point for the fly was discarded from further analysis. Otherwise, the log ratio of anterior grooming to posterior grooming was calculated as following:

$${\text{Log}}_{10}(\text{ Anterior grooming Posterior grooming })={\text{log}}_{10}\left\{[\text{ Frame number (anterior grooming) }+1]/[\text{Frame number}(\text{posterior grooming})+1]\right\}$$

## DATA AND CODE AVAILABILITY

Ethogram data, modeling code and an example video for fly-on-a-ball experiment are available at https://data.mendeley.com/datasets/fxz8dgywcd/1.

## Supplementary Material

2

## Figures and Tables

**Table:** KEY RESOURCES TABLE

REAGENT or RESOURCE	SOURCE	IDENTIFIER
Antibodies		
chicken polyclonal to GFP	Abcam	Cat#13970
mouse monoclonal brp antibody	DSHB	Cat#AB_2314866
anti-chicken Alexa Fluor 488	Invitrogen	Cat#A-11039
anti-mouse Alexa Fluor 633	Invitrogen	Cat#A-21052
Chemicals, Peptides, and Recombinant Proteins		
Reactive Yellow 86	Organic Dyestuffs Corporation	CAS 61951–86-8
Insect-a-slip	BioQuip Products	Cat#2871A
UV glue	Bondic	N/A
Experimental Models: Organisms/Strains		
*Canton S*	Bloomington Stock Center	RRID: BDSC_64349
*Control-spGAL4: BPp65ADZp (attP40); BPZpGDBD (attP2)*	Bloomington Stock Center	RRID: BDSC_79603
*Bristle-spGAL4–1: R38B08-AD; R81E10-DBD*	Bloomington Stock Center	RRID: BDSC_71032; RRID: BDSC_68529
*Bristle-spGAL4–2: R38B08-AD; R70C11-DBD*	Bloomington Stock Center	RRID: BDSC_71032; RRID: BDSC_70292
*Wing+haltere CS-spGAL4: R83H05-AD; R31H10-DBD*	Bloomington Stock Center	RRID: BDSC_68688; RRID: BDSC_69835
*Control-GAL4: pBDPGal4U*	Bloomington Stock Center	RRID: BDSC_68384
*CO-GAL4: iav-GAL4*	Bloomington Stock Center	RRID: BDSC_52273
*SR-GAL4: stum-GAL4*	Bloomington Stock Center	RRID: BDSC_58777
*HP-GAL4: R48A07-GAL4*	Bloomington Stock Center	RRID: BDSC_50340
*MD-GAL4: ppk-GAL4*	Bloomington Stock Center	RRID: BDSC_32079
*Eye bristle-spGAL4: R38B08-AD; VT043775-DBD*	Bloomington Stock Center	RRID: BDSC_71032; RRID: BDSC_73728
*Antennal CO-spGAL4: R61D08-AD; R27H08-DBD*	Bloomington Stock Center	RRID: BDSC_71105; RRID: BDSC_69106
*R74C07-GAL4*	Bloomington Stock Center	RRID: BDSC_39847
*R52A06-GAL4*	Bloomington Stock Center	RRID: BDSC_38810
*R21D12-GAL4*	Bloomington Stock Center	RRID: BDSC_48946
*R73D10-GAL4*	Bloomington Stock Center	RRID: BDSC_39819
*R86D09-GAL4*	Bloomington Stock Center	RRID: BDSC_40459
*VT028607-GAL4*	Vienna Drosophila Resource Center	Cat#203789
*R14F05-GAL4*	Bloomington Stock Center	RRID: BDSC_49257
*Gr33a-GAL4*	Bloomington Stock Center	RRID: BDSC_31425
*Gr64f-GAL4*	Bloomington Stock Center	RRID: BDSC_57669
*ppk28-GAL4*	[50]	N/A
*Or56a-GAL4*	Bloomington Stock Center	RRID: BDSC_23896
*Orco-GAL4*	Bloomington Stock Center	RRID: BDSC_23292
*Control-LexA: pBDPLexAp65U (attP40)*	Bloomington Stock Center	RRID: BDSC_77691
*R42G12-LexA*	Bloomington Stock Center	RRID: BDSC_53643
*20XUAS-CsChrimson-mVenus (attp18)*	Bloomington Stock Center	RRID: BDSC_55134
*20XUAS-ChrimsonR-mCherry (attp18)*	[30]	N/A
*13XLexAop2-CsChrimson-mVenus (attp18)*	Bloomington Stock Center	RRID: BDSC_55137
*10XUAS-IVS-eGFPKir2.1 (attP2)*	[51]	N/A
*10XUAS-IVS-mCD8::GFP (attP2)*	Bloomington Stock Center	RRID: BDSC_32185
*P[sev-wg, w*^*-*^*]*	[32]	N/A
*eya*^*2*^	Bloomington Stock Center	RRID: BDSC_2285
*so*^*D*^	Bloomington Stock Center	RRID: BDSC_4287
*orco*^*1*^	Bloomington Stock Center	RRID: BDSC_23129
*orco*^*2*^	Bloomington Stock Center	RRID: BDSC_23130
Software and Algorithms		
Adobe Illustrator	https://www.adobe.comproducts/illustrator.html/	RRID:SCR_010279
MATLAB	http://www.mathworks.com/products/matlab/	RRID:SCR_001622
Python	http://www.python.org/	RRID:SCR_008394
Fiji	http://fiji.sc/	RRID:SCR_002285
VCode	http://social.cs.uiuc.edu/projects/vcode.html	N/A
Automatic Behavior Recognition System (ABRS)	[15]	N/A

## Highlights

- Multiple mechanosensory neurons induce grooming; other senses are not required
- Only activation of distributed bristle neurons results in A → P sequence
- Grooming hierarchy is determined by ratio of sensory inputs to different body parts
- A → P progression depends on changes in ratio of sensory inputs over time
