# Spatial Comparisons of Mechanosensory Information Govern the Grooming Sequence in *Drosophila*

**DOI:** 10.1016/j.cub.2020.08.070

**Published:** 2020-09-21

**Authors:** Neil Zhang, Li Guo, Julie H. Simpson

During figure generation, the lower panels of Figures S2E and S2F were switched. We checked the original confocal images and restained our R86D09-GAL4 stock to confirm where the error occurred. It does not alter the behavioral analysis or conclusions of the paper. The corrected version of the figure has been published online. In addition, some reference numbers in the STAR Methods were incorrect and we omitted two references; this is now fixed as well. The authors apologize for the errors.

## Figures and Tables

**Figure S2.**
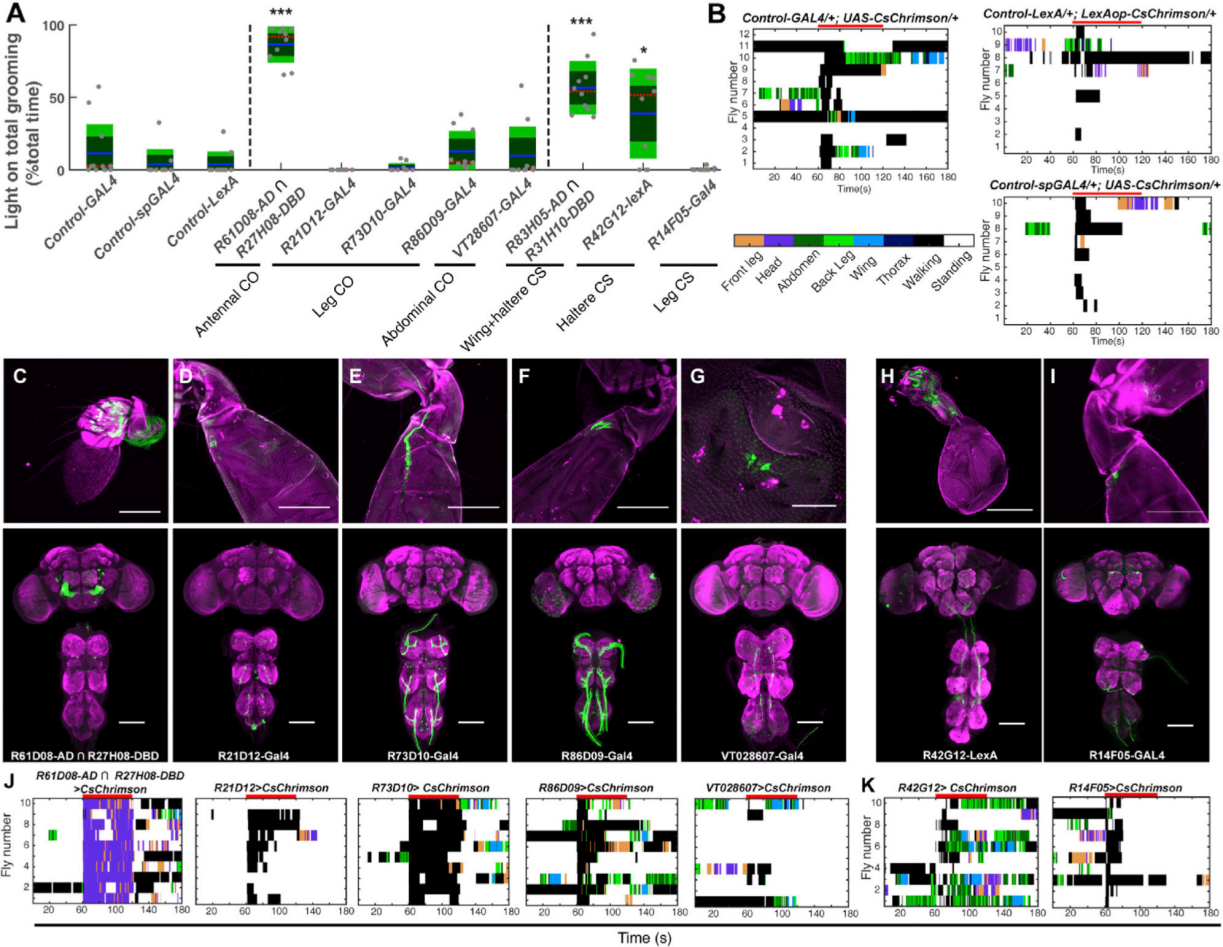
Grooming can be induced by subsets of chordotonal organs and campaniform sensilla on different body parts. Related to Figure 1. (corrected)

**Figure S2.**
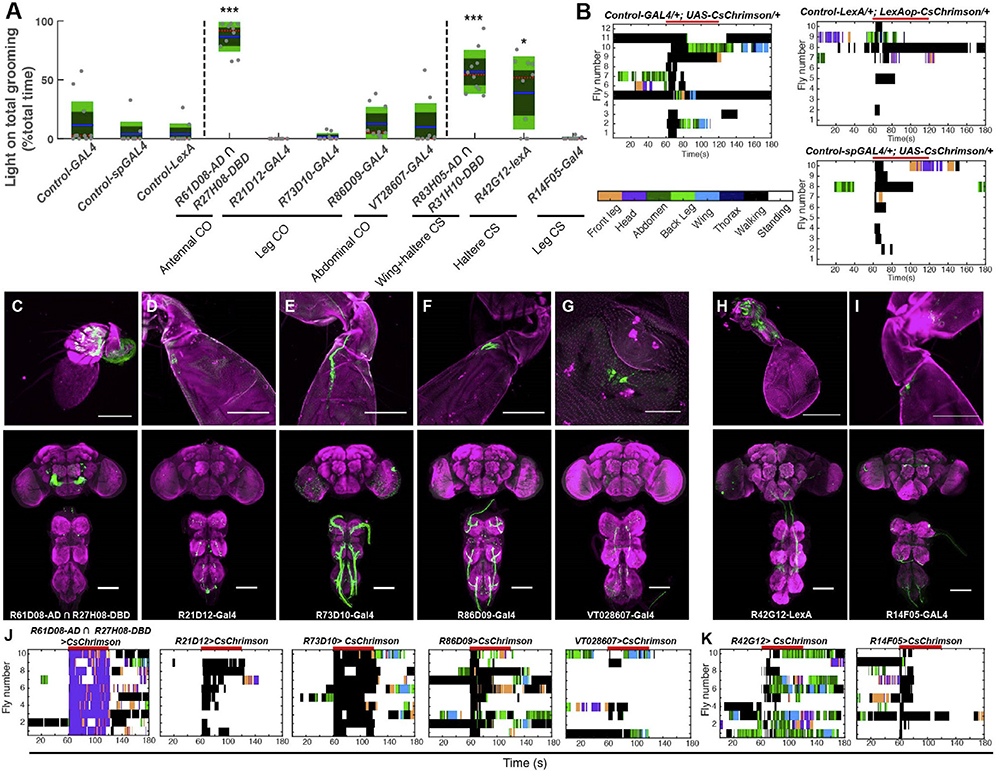
Grooming can be induced by subsets of chordotonal organs and campaniform sensilla on different body parts. Related to Figure 1. (original)

