# Supplementary material for: Spatial Comparisons of Mechanosensory Information Govern the Grooming Sequence in Drosophila
Source: Curr Biol. Author manuscript; Available in PMC 2021 Mar 23. (PMC7184881; doi:10.1016/j.cub.2020.01.045)
Supplement: 2 [file NIHMS1569186-supplement-2.pdf]

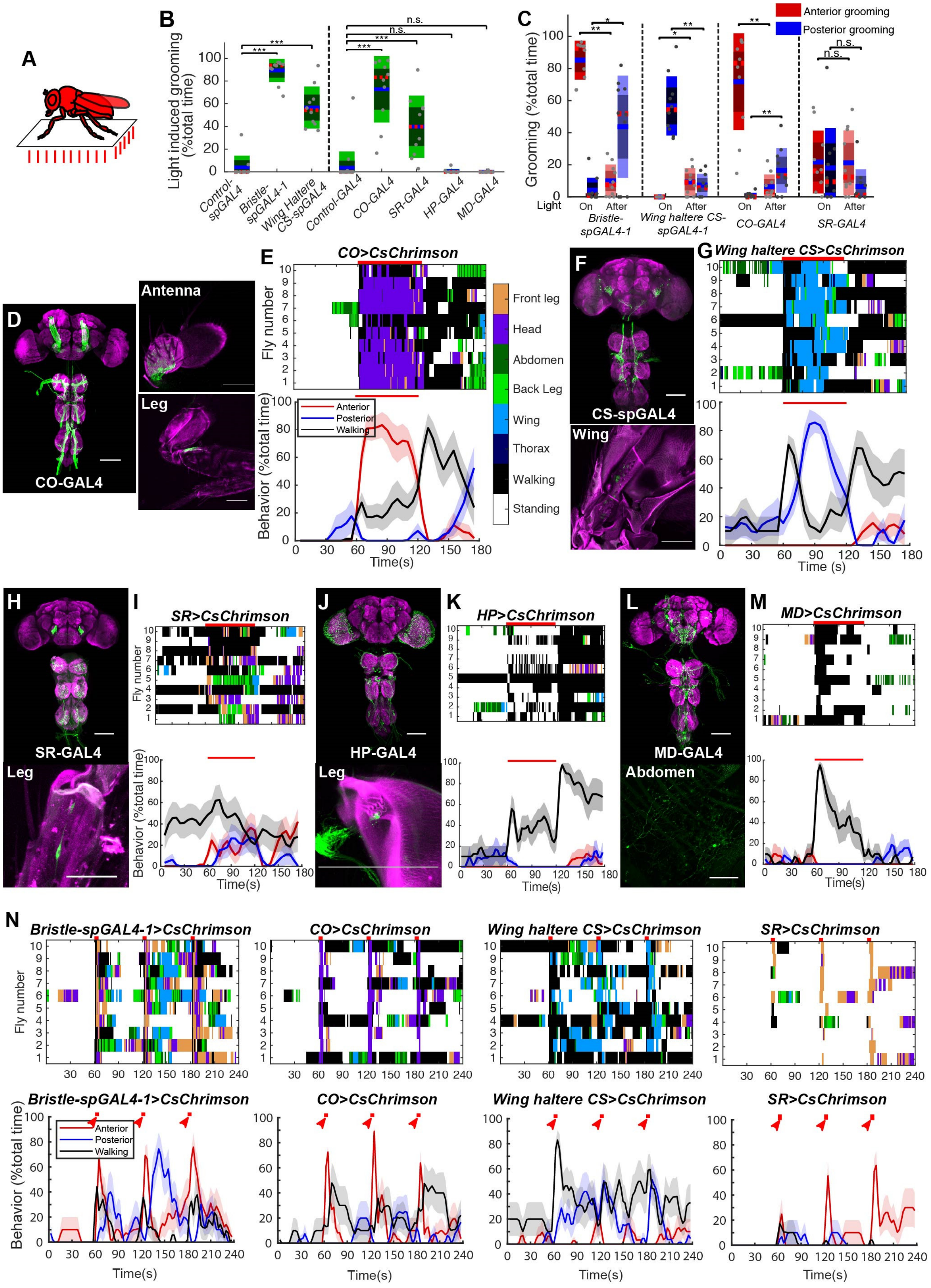
A
B
Light induced grooming (%total time)
Control-spGAL4
Bristle-spGAL4-1
Wing Haltere CS-spGAL4
Control-GAL4
CO-GAL4
SR-GAL4
HP-GAL4
MD-GAL4
***
n.s.
C
Grooming (%total time)
Anterior grooming
Posterior grooming
Light
On
After
Bristle-spGAL4-1
Wing haltere CS-spGAL4-1
CO-GAL4
SR-GAL4
*
**
n.s.
D
CO-GAL4
Antenna
Leg
E
CO>CsChrimson
Fly number
Front leg
Head
Abdomen
Back Leg
Wing
Thorax
Walking
Standing
Anterior
Posterior
Walking
Behavior (%total time)
Time(s)
F
CS-spGAL4
Wing
G
Wing haltere CS>CsChrimson
Time (s)
H
SR-GAL4
Leg
I
SR>CsChrimson
J
HP-GAL4
Leg
K
HP>CsChrimson
L
MD-GAL4
Abdomen
M
MD>CsChrimson
N
Bristle-spGAL4-1>CsChrimson
CO>CsChrimson
Wing haltere CS>CsChrimson
SR>CsChrimson
Time(s)

Figure S1. Grooming behavior can be induced by different mechanosensory neurons and different optogenetic stimulation protocols. Related to Figure 1.

(**A**) Experiment setup. Light was delivered below to freely-moving flies. (**B**) The percent of time flies spent in grooming during 1-minute optogenetic activation of different groups of mechanosensory neurons ($n \geq 10$). CO: chordotonal organ. CS: campaniform sensilla. SR: stretch receptor. HP: hair plate. MD: multidendritic neurons. The GAL4 and splitGAL4 lines used are listed in the Key Resources Table. (**C**) Anterior grooming and posterior grooming are quantified during (60-120s) and after (120-180s) light stimulus. (**D**, **F**, **H**, **J**, **L**) The expression pattern of driver lines in central nervous system and peripheral nervous system. (**E**, **G**, **I**, **K**, **M**) Grooming response induced by light activation of different groups mechanosensory neurons. Optogenetic stimulation is indicated by red line. Data is plotted as descripted in **Figure 1G**, **H.** (**N**) Grooming response induced by 5s optogenetic stimulation at 60s, 120s and 180s. Behavior probabilities over total time are calculated every 2.5 seconds in a sliding 5-second time window.

Kruskal-Wallis test with Wilcoxon rank-sum post hoc (**B**) or Wilcoxon signed-rank test (**C**) were used for significance tests. Asterisks represent the following p values: $*p < 0.05$, $**p < 0.01$, $***p < 0.001$.

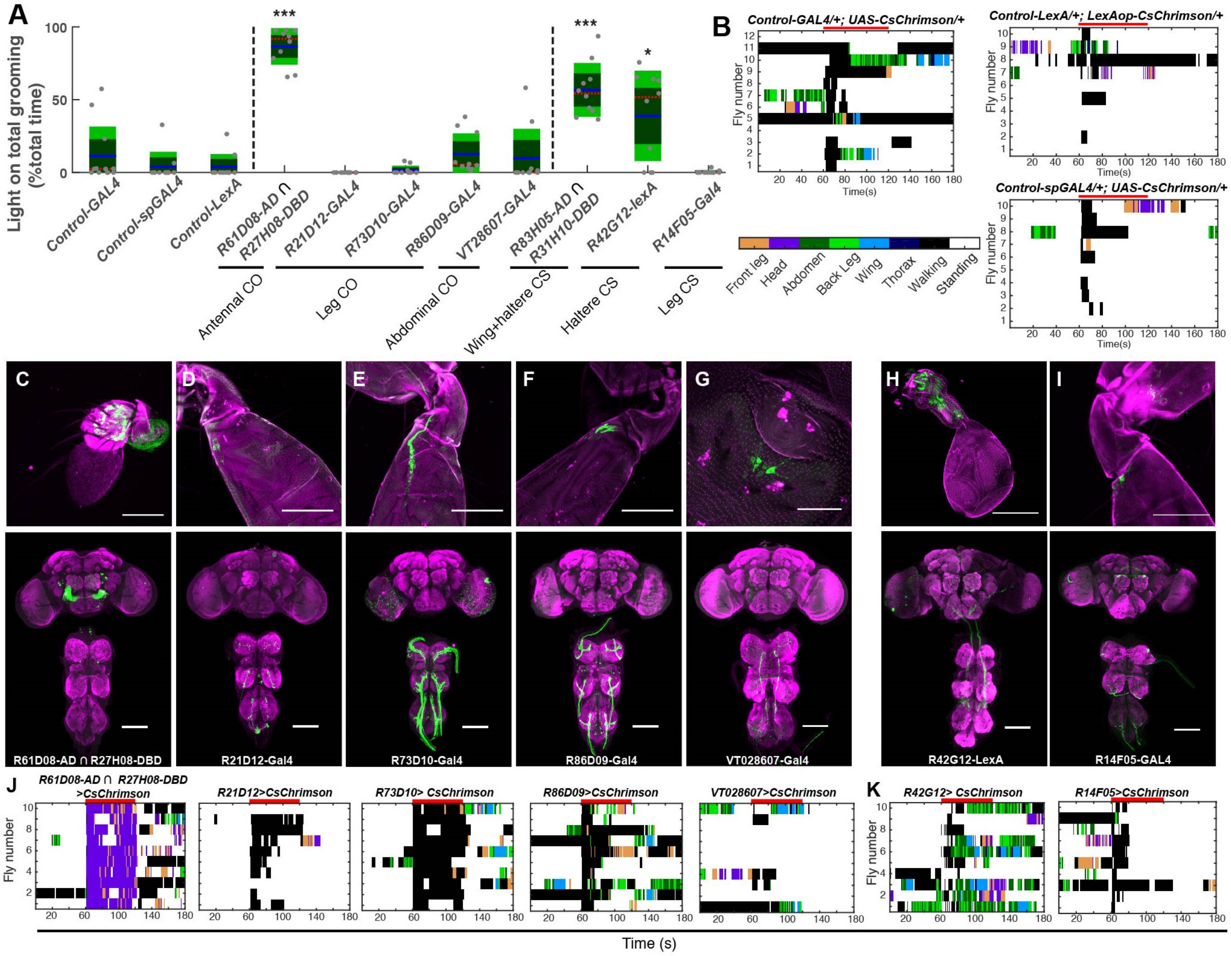

Figure S2. Grooming can be induced by subsets of chordotonal organs and campaniform sensilla on different body parts. **Related to Figure 1.**

(**A**) The percent of total grooming induced by red light sensitive CsChrimson expressed in chordotonal organs or campaniform sensilla (n≥10). Wilcoxon rank-sum test was used to compare each line with the corresponding control line. Asterisks represent the following p values: $*p < 0.05$, $**p < 0.01$, $***p < 0.001$. (**B**) Grooming ethograms of optogenetic stimulation in control flies. Red solid lines represent 1-minute light stimulus. (**C**-**I**) Expression patterns of driver lines targeting antennal (C), leg (D-F) and abdominal (G) chordotonal organs and haltere (H), leg (I) campaniform sensilla are visualized with *UAS-mCD8-GFP* in peripheral sensory organs and central nervous system. Magenta represents cuticle autofluorescence in peripheral sensory organs and neuropil (nc82 antibody) in central nervous system. Scale bars, 100μm. (**J**) Grooming ethograms of flies with optogenetic activation of different chordotonal organs. Red solid lines represent 1-minute light stimulus. (**K**) Grooming ethograms of flies with optogenetic activation of different campaniform sensilla. The expression pattern and grooming response of *Wing haltere CS-spGAL4* are shown in **Figure S1F, G**.

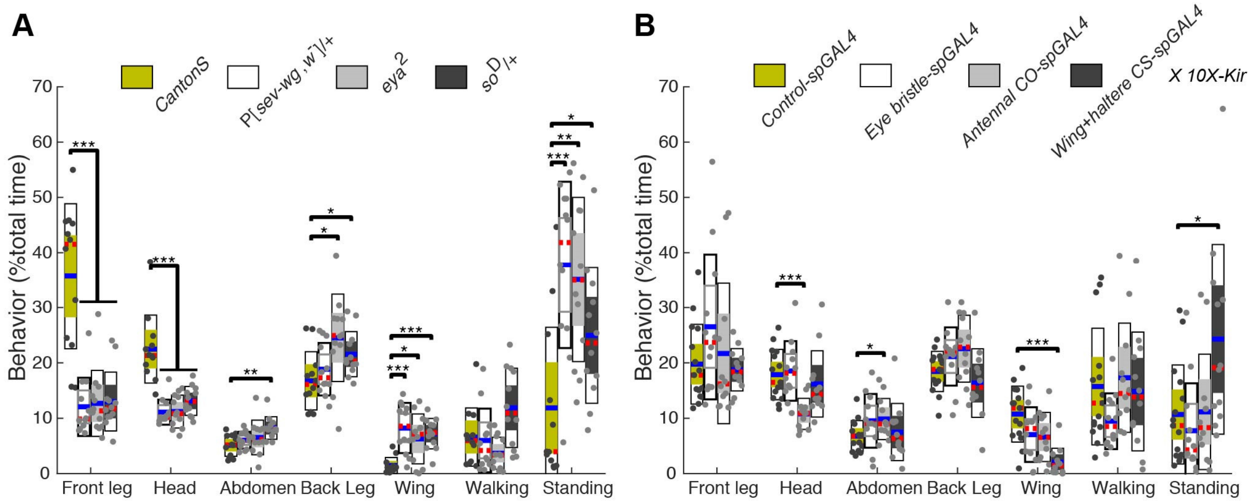

Figure S3. Loss of activity in sensory organs on specific body parts only disrupts grooming of that part. Related with Figure 2.

Histograms show the percent of time that mutants lack eye bristles (A) or flies with constitutive inhibition of specific mechanosensory neurons (B) performed each behavior in dusting experiments (n≥12).

Kruskal-Wallis test with Wilcoxon rank-sum post hoc were used for significance tests. Asterisks represent the following p values: *p < 0.05, **p < 0.01, ***p < 0.001.

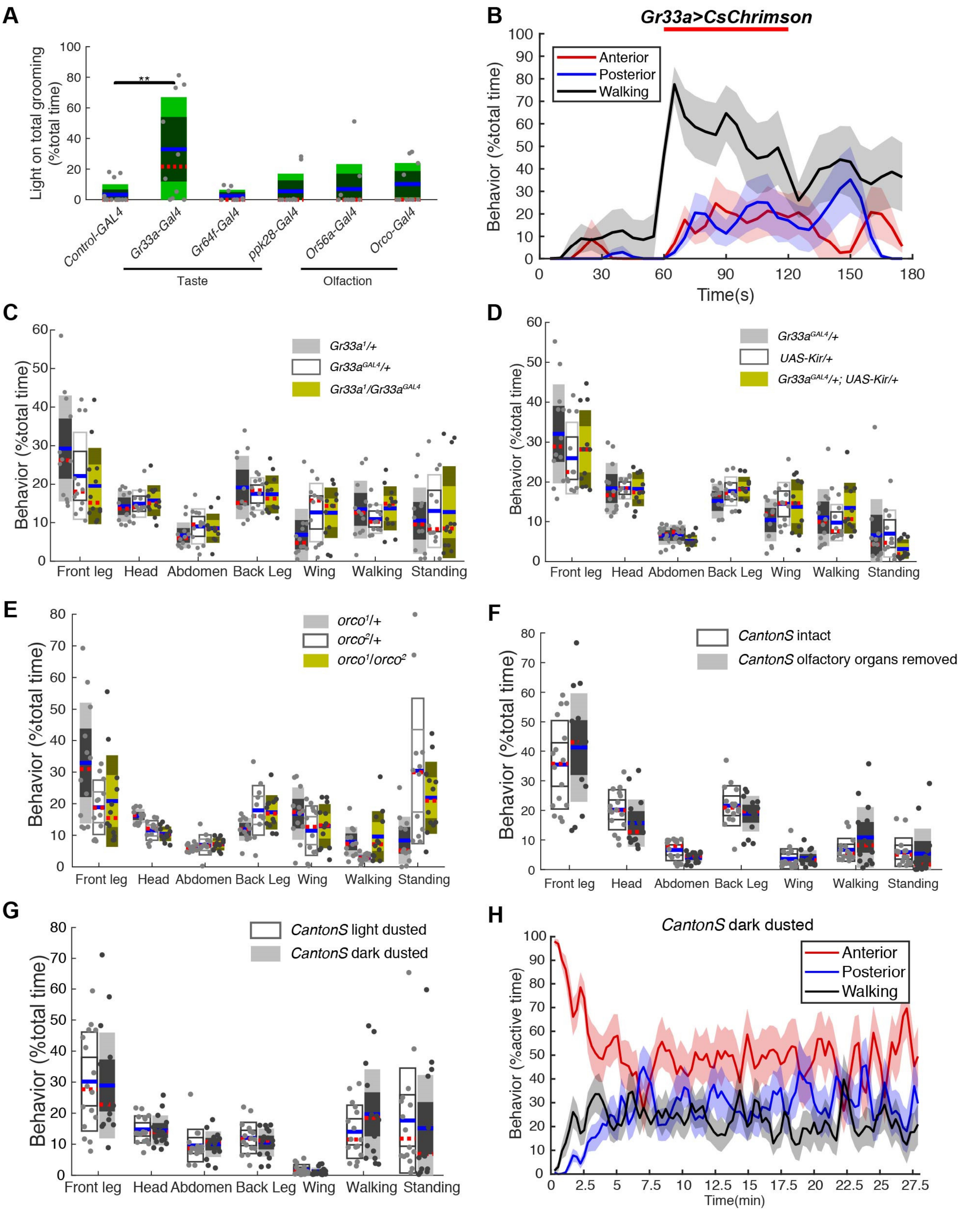

Figure S4. Taste, olfaction and vision are not necessary for dust-induced grooming. Related to Figures 1 and 2.

(**A**) The percent of total grooming induced by 1-minute light activation to CsChrimson-expressing taste or olfactory neurons (n≥10). (**B**) Behavior probabilities induced by optogenetic activation of bitter taste neurons. Red solid lines represent 1-minute light stimulus. Behavior probabilities at different time points are quantified as in **1H**. (**C**) Percent of time each behavior performed by dusted *Gr33a* null-mutant heterozygotes and control flies (n=12). No significant change was found between mutant and both controls. (**D**) Constitutive inhibition of bitter taste neurons did not change the amount of time flies spent performing each grooming movement (n≥11). (**E**) Percent of time each behavior was performed by dusted *orco* null-mutant homozygotes and control flies (n=12). No significant change was found. (**F**) Percent of time each behavior was performed by dusted *Canton S* with or without olfactory organs (n=12). No significant change was found. (**G**) Grooming in the light or dark did not change the amount of time flies spent performing each grooming movement (n≥12). (**H**) Behavior probabilities for dusted flies grooming in dark. Behavior probabilities at different time points are quantified as in **1D**.

Wilcoxon rank-sum test was used for significance tests. Asterisks represent the following p values: *$p < 0.05$, **$p < 0.01$, ***$p < 0.001$.

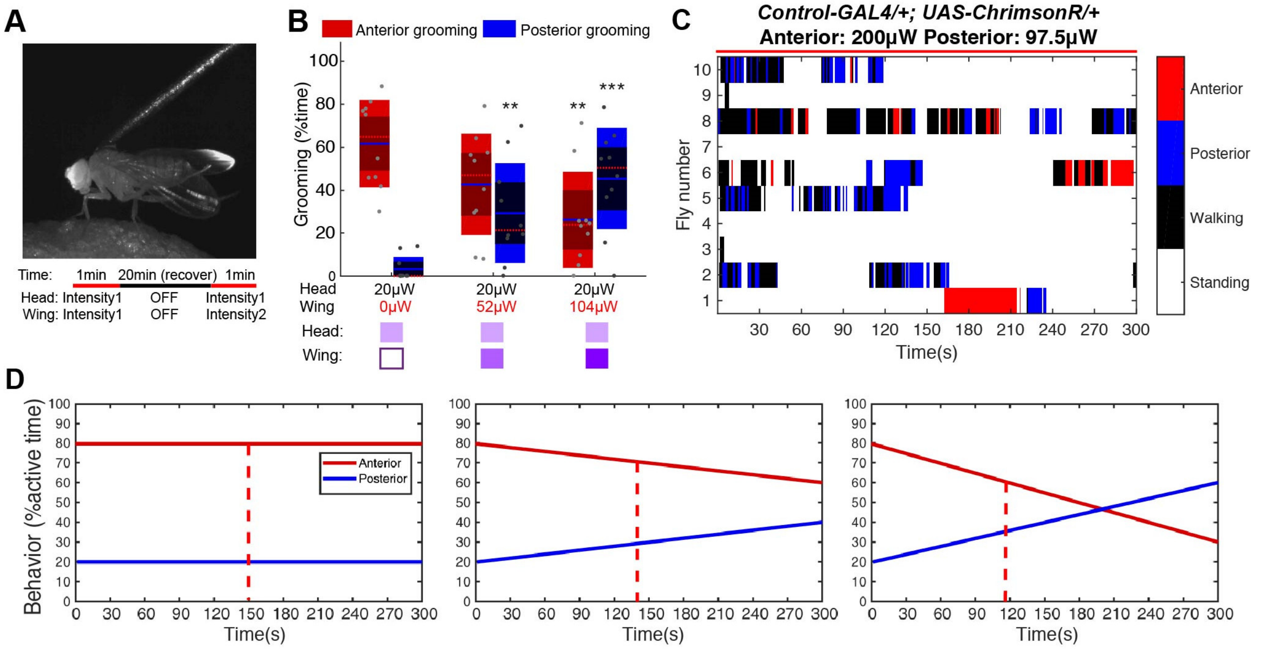

Figure S5. Investigating grooming hierarchy and progression through "fly-on-a-ball" system. Related to Figures 3-6.

(**A**) Photo of "fly-on-a-ball" system with competition between head and wings. Two light fibers target head and wings separately. Each fly was tested in two different 1-minute light stimulations. 20 minutes recovery time was given between the two stimulations. (**B**) In tethered *R74C07>ChrimsonR* flies, light on the head was kept constant while light on the wings was increased in different experiments, a decreased ratio of anterior to posterior grooming was observed (n=10). Wilcoxon rank-sum test was used. Grooming time induced by each light condition was compared with the first group. Asterisks represent the following p values: *$p < 0.05$, **$p < 0.01$, ***$p < 0.001$. (**C**) Light stimulation of control flies in "fly-on-a-ball" system did not induce obvious grooming response. Optogenetic stimulation is indicated by red line. (**D**) Diagram shows that the "anterior grooming half-time" serves as a measure of progression speed. The anterior grooming half-time is the time point when flies finish half of the total anterior grooming. Flies that progress to posterior grooming faster reach half-time (indicated by red dashed line) sooner.

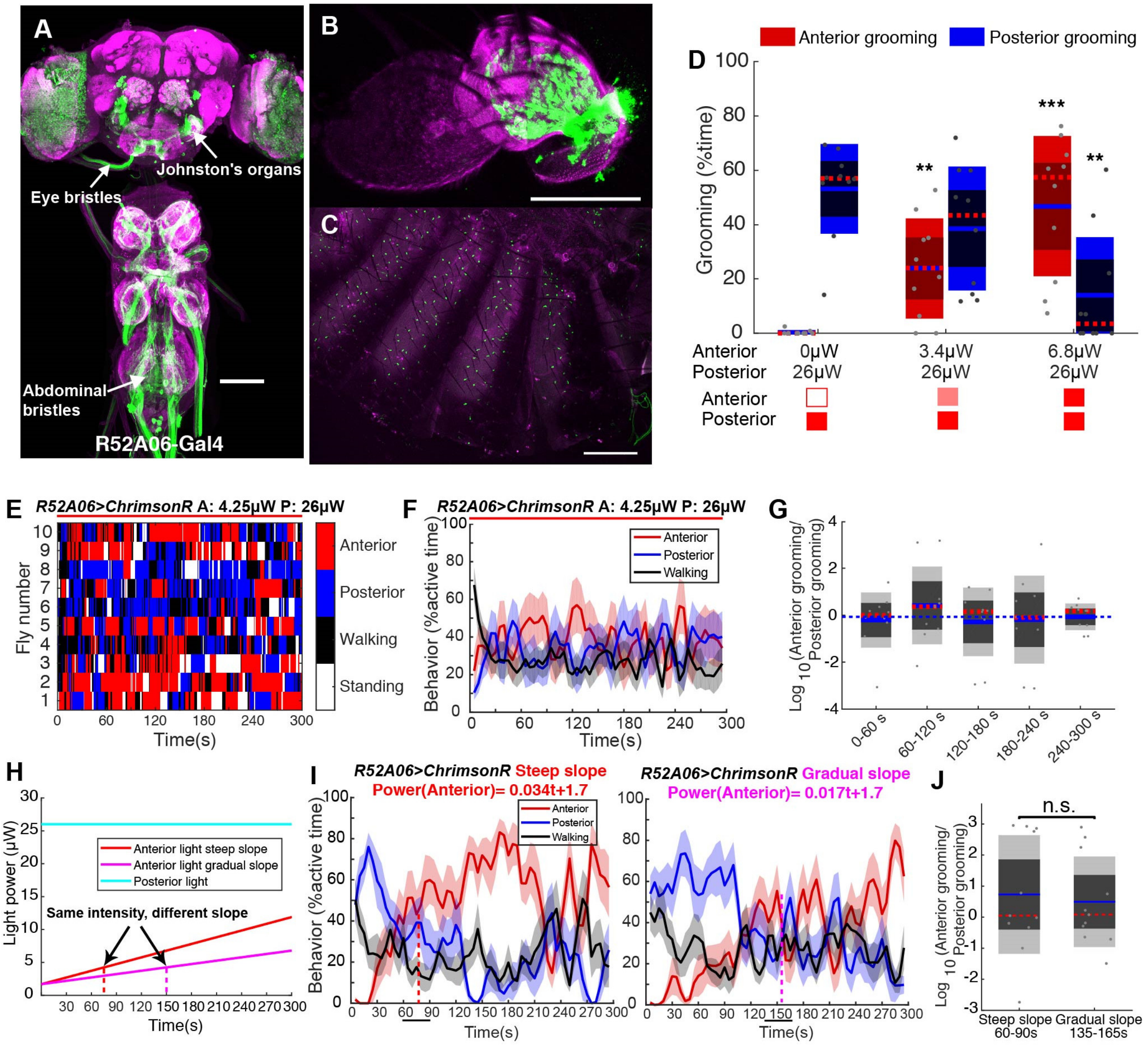

Figure S6. The importance of spatial comparison for grooming sequence is confirmed with a different mechanosensory neuron driver. Related to Figures 3, 4 and 6.

(**A**) Expression patterns of R52A06-GAL4 which targets Johnston's organ neurons (**B**) and eye bristle neurons on the head and bristle neurons in the abdomen (**C**). Magenta represents cuticle autofluorescence in peripheral sensory organs and neuropil (nc82 antibody) in central nervous system. Scale bars, 100µm. (**D**) Grooming response induced by different 1min optogenetic activation in tethered *R52A06>ChrimsonR* flies. Same protocols are used as in **3H** (n=10). (**E-G**) In tethered *R52A06>ChrimsonR* flies, constant level anterior and posterior light stimulus was given for 5 minutes (n=10). (**E**) Ethograms of tested flies. Behavior probabilities (**F**) and anterior to posterior grooming ratio (**G**) is quantified as in **4B**. No significant difference was found among different time windows. (**H-J**) Tethered *R52A06>ChrimsonR* flies were tested in two light conditions, same constant light was given to posterior body part; anterior light crossed the equilibrium point (4.25µW:26µW, indicated by arrow) at different slopes and times (n=11). (**I**) Behavior probabilities at different time points are quantified as in **4B**. (**J**) The ratio of anterior grooming to posterior grooming within the 30 s time window around the target light intensity point.

Kruskal-Wallis test with Wilcoxon rank-sum post hoc (**D**), Kruskal-Wallis test (**G**) or Wilcoxon signed-rank test (**J**) were used for significance tests. Asterisks represent the following p values: $^{*}p < 0.05$, $^{**}p < 0.01$, $^{***}p < 0.001$.
